# Phylogenomic and genomic analysis reveals unique and shared genetic signatures of Mycobacterium kansasii complex species

**DOI:** 10.1099/mgen.0.001266

**Published:** 2024-07-17

**Authors:** Edson Machado, Sidra Vasconcellos, Lia Gomes, Marcos Catanho, Jesus Ramos, Luciana de Carvalho, Telma Goldenberg, Paulo Redner, Paulo Caldas, Carlos Campos, Margareth Dalcolmo, Maria Cristina Lourenço, Elena Lasunskaia, Vinicius Mussi, Lizania Spinassé, Solange Vinhas, Leen Rigouts, Sari Cogneau, Pim de Rijk, Christian Utpatel, Jarmila Kaustova, Tridia van der Laan, Han de Neeling, Nalin Rastogi, Klavdia Levina, Marge Kütt, Igor Mokrousov, Viacheslav Zhuravlev, Ndivhu Makhado, Manca Žolnir-Dovč, Vera Jankovic, Jacobus de Waard, Maria Carolina Sisco, Dick van Soolingen, Stefan Niemann, Bouke C. de Jong, Conor J. Meehan, Philip Suffys

**Affiliations:** 1 Laboratório de Biologia Molecular Aplicada a Micobactérias, Instituto Oswaldo Cruz, Fiocruz, Rio de Janeiro, RJ, Brazil; 2 Laboratório de Genética Molecular de Microrganismos, Instituto Oswaldo Cruz, Fiocruz, Rio de Janeiro, RJ, Brazil; 3 Laboratório de Referência Nacional para Tuberculose, Centro de Referência Professor Hélio Fraga, Escola Nacional de Saúde Pública, Fiocruz, Rio de Janeiro, RJ, Brazil; 4 Serviço de Pesquisa Clínica, Centro de Referência Professor Hélio Fraga, Escola Nacional de Saúde Pública, Fiocruz, Rio de Janeiro, RJ, Brazil; 5 Laboratório de Bacteriologia e Bioensaios, Instituto Nacional de Infectologia, Fiocruz, Rio de Janeiro, RJ, Brazil; 6 Laboratório de Biologia do Reconhecer, Universidade Estadual do Norte Fluminense Darcy Ribeiro, Campos dos Goytacazes, RJ, Brazil; 7 Núcleo de Doenças Infecciosas, Universidade Federal do Espírito Santo, Vitória, ES, Brazil; 8 Unit of Mycobacteriology, Institute of Tropical Medicine, Antwerp, Belgium; 9 Molecular and Experimental Mycobacteriology, Research Center Borstel, Borstel, Germany; 10 German Center for Infection Research, Partner Site Hamburg-Lübeck-Borstel-Riems, Borstel, Germany; 11 Department of Diagnostic Mycobacterioses, Regional Institute of Public Health, Ostrava, Czech Republic; 12 National Institute for Public Health and the Environment, Bilthoven, Netherlands; 13 TB and Mycobacteria Unit, Institut Pasteur de Guadeloupe, Guadeloupe, France; 14 Mycobacteriology Section of Microbiology Laboratory, North Estonia Medical Centre, Tallinn, Estonia; 15 Laboratory of Molecular Epidemiology and Evolutionary Genetics, St. Petersburg Pasteur Institute, St. Petersburg, Russia; 16 St. Petersburg Research Institute of Phthisiopulmonology, St. Petersburg, Russia; 17 Department of Microbiological Pathology, Sefako Makgatho Health Sciences University, Pretoria, South Africa; 18 National Health Laboratory Service, Dr George Mukhari Tertiary Laboratory, Medical Microbiology, Pretoria, South Africa; 19 Global Institute of Health, University of Antwerp, Antwerp, Belgium; 20 National Reference Laboratory for Mycobacteria, University Clinic of Respiratory and Allergic Diseases, Golnik, Slovenia; 21 Mycobacteria Reference Laboratory, Croatian National Institute of Public Health, Zagreb, Croatia; 22 Tuberculosis Department. Servicio Autónomo Instituto de Biomedicina Dr. Jacinto Convit, Universidad Central de Venezuela, Caracas, Venezuela; 23 One Health Research Group, Universidad de Las Américas, Quito, Ecuador; 24 Department of Biosciences, Nottingham Trent University, Nottingham, UK

**Keywords:** lineages, *Mycobacterium kansasii*, pangenome, phylogenomics, virulence

## Abstract

Species belonging to the *Mycobacterium kansasii* complex (MKC) are frequently isolated from humans and the environment and can cause serious diseases. The most common MKC infections are caused by the species *M. kansasii* (*sensu stricto*), leading to tuberculosis-like disease. However, a broad spectrum of virulence, antimicrobial resistance and pathogenicity of these non-tuberculous mycobacteria (NTM) are observed across the MKC. Many genomic aspects of the MKC that relate to these broad phenotypes are not well elucidated. Here, we performed genomic analyses from a collection of 665 MKC strains, isolated from environmental, animal and human sources. We inferred the MKC pangenome, mobilome, resistome, virulome and defence systems and show that the MKC species harbours unique and shared genomic signatures. High frequency of presence of prophages and different types of defence systems were observed. We found that the *M. kansasii* species splits into four lineages, of which three are lowly represented and mainly in Brazil, while one lineage is dominant and globally spread. Moreover, we show that four sub-lineages of this most distributed *M. kansasii* lineage emerged during the twentieth century. Further analysis of the *M. kansasii* genomes revealed almost 300 regions of difference contributing to genomic diversity, as well as fixed mutations that may explain the *M. kansasii*’s increased virulence and drug resistance.

**Impact Statement**

*Mycobacterium kansasii* complex (MKC) is a group of closely related non-tuberculous mycobacteria species, recognized as a significant source of human infection. Species belonging to the MKC may present a broad spectrum of virulence, antimicrobial resistance and pathogenicity and there is a lack of knowledge about their genomic content related to these broad phenotypes. We have provided whole genomic sequencing DNA for 342 MKC isolates and, together with public data, investigated the MKC pangenome, mobilome, resistome, virulome and defence systems and show unique and shared genetic signatures within MKC species. Furthermore, with phylogenomic and Bayesian population analysis, we inferred the distribution and emergence of the *Mycobacterium kansasii* species lineages and sub-lineages. This study has considerably expanded the MKC available data, by providing genomic sequences of isolates from countries and global regions with unknown or poorly described MKC data until then.

## Data Summary

Next generation sequencing (NGS) data generated in the study are available in the NCBI Sequence Read Archive (SRA) repository under the accession number PRJNA1048499 and the accession numbers for all data sets used are provided in Table S1 (available in the online version of this article).

## Data Availability

All sequenced reads are available in BioProject PRJNA1048499.

Supplementary tables 1 to 19 providing details of used data and results of this study are available on the Microbiology Figshare data repository platform: https://doi.org/10.6084/m9.figshare.26014465.v1 [1].

## Introduction

The *Mycobacterium kansasii* complex (MKC) is a group of closely related non-tuberculous *Mycobacterium* (NTM) species. Currently, the MKC includes *Mycobacterium gastri* and six *Mycobacterium kansasii* subtypes that were recently redefined as distinct species based on genomic taxonomy and phylogenomic analyses [2,3]. Thus, the former *M. kansasii* subtypes I to VI were renamed as *Mycobacterium kansasii* (*sensu stricto*)*, Mycobacterium persicum, Mycobacterium pseudokansasii, Mycobacterium ostraviense, Mycobacterium innocens,* and *Mycobacterium attenuatum*.

In the clinical niche, *M. kansasii* is the most common MKC species isolated, followed by *M. persicum*, which is mainly associated with individuals infected by human immunodeficiency virus (HIV), whereas other MKC species members remain predominantly colonisers and rarely associated with disease [4]. Similar to some other NTM species, strains of the MKC can also be isolated from animal and environmental sources. Besides the pathogenicity and isolation source diversity, MKC species display heterogeneity regarding phenotypic aspects such as virulence, colony morphology and drug resistance. For example, in regard to virulence, it was observed that lung disease caused by *M. kansasii* strains can result in a broad pathology spectrum [5].

At the genomic level, the basis of the MKC pathogenicity and phenotypic traits has been explored. Plasmids are estimated to be present in around 30 % of MKC strains, although with no observed correlation with pathogenicity [6]. Conversely, an association of pathogenicity with genetic recombination has been observed, mediated by distributive conjugal transfer, also with possible correlation to MKC speciation events [6,7]. Regarding virulence, whereas *M. kansasii* contains a type-VII secretion system ESX-1 locus with all corresponding orthologs of the *M. tuberculosis* genes, some MKC species do not have an intact conservation of this locus [6,8]. Furthermore, nearly three hundred shared orthologs between *M. kansasii* and *M. tuberculosis* are predicted to encode potential virulence factors [5] (VFs), but the conservation of these VFs in another MKC species has not been explored.

Concerning the resistance of members of the MKC to antimicrobial agents, estimates are variable, but some recent studies conducted in China [9] and Brazil [10] estimated a high percentage (~70 %) of *M. kansasii* isolates resistant to ethambutol and ciprofloxacin, while between 10 and 20 % of the isolates were resistant to rifampicin. However, in *M. kansasii*, no clear relationship has been observed between mutations in particular genes and drug resistance as observed in *M. tuberculosis* [11].

Due to the higher interest in isolating MKC from clinical rather that from environmental sources, and the strong association of *M. kansasii* with pulmonary disease, much more genomic sequencing data are available from the latter species, with the majority of data coming from Europe, China, and Australia leaving knowledge of other geographical areas lacking [7,9]. From *M. kansasii* genomic data, it was estimated that some lineages might be globally spread [7], while genomes obtained from clinical isolates in Brazil and China [9,12] suggested at least four genetically diverse groups.

Even though some questions about the MKC pathogenicity, virulence, drug resistance, genetic diversity, and epidemiology have been addressed by analysing genomic data [2,3, 5,9, 11], several aspects about the MKC species genomic characteristics and epidemiology remains unexplored. For instance, (i) in addition to plasmids, the prevalence and characteristics of mobile genetic elements in MKC genomes, such as prophages; (ii) the possible plasmids or prophages contribution to virulence or drug resistance; (iii) the MKC pangenome structure and diversity; (iv) the MKC viral defence mechanisms repertoire; (v) the potential *M. kansasii* lineages and their association with certain geographical areas or niches; and vi) transmission mechanisms of the different MKC species.

We have therefore sequenced the genomes of nearly four hundred MKC isolates and together with publicly available data, performed several *in silico* analyses to further address the opened questions mentioned above. In this study we provide a global phylogeny and a genomic panorama of the MKC, that provides new knowledge about the MKC mobilome, resistome, virulome, pangenome, viral defence systems, population structure and genetic diversity.

## Methods

### Data, sample collection, and whole-genome sequencing

The present study includes a total of 342 isolates from 25 different countries, encompassing five continents. Briefly, 298 isolates were obtained from human clinical samples, 45 from environmental niches, 41 with unknown origin, and four from animals, collected between 1996 to 2016. These isolates are derived from culture colonies presenting MKC-like phenotypic characteristics and were subjected to whole genome sequencing. NGS libraries were constructed from genomic DNA using a modified Nextera protocol [14] and sequenced on the Illumina NextSeq 500 platform with 2×151 bp runs (Illumina, San Diego, CA, USA). We additionally sequenced with PacBio long-read sequencing on a Sequel II instrument, using the microbial SMRTbell 2.0 workflow (Pacific Biosciences, Menlo Park, CA, USA) three MKC isolates (KAN-C06235, KAN-130495 and KAN-960446) to generate complete or near fully complete closed genome sequences, as these strains had enough DNA of sufficient quality for PacBio sequencing, in order to decipher their plasmid content. In addition to our data collection, we retrieved available MKC sequence read data sets and assembled genomes from NCBI nucleotide databases as of May 2021, by searching for ‘*Mycobacterium kansasii*’ term and excluding NGS data from non-Illumina platforms. Public sequence read data was downloaded from SRA database and converted into FASTQ files using the NCBI SRAtoolkit (v2.10.8, https://ncbi.github.io/sra-tools/).

### Genome assembly and annotation

Illumina sequencing reads were trimmed and filtered using Trimmomatic (v0.36) [15] and genomes were assembled with SPAdes (v3.14.1) [16] applying the careful mode, auto coverage cutoff, mismatch corrections, and automatic selection of the k-mers size based on read length. Contigs of less than 500 bp and coverage lower than 2× were discarded. PacBio genomes were assembled with CANU (release 10117) [17] applying the following parameters: *genomeSize=6.57* mb for all samples, *minReadLength=2000* for sample KAN-960446, *minReadLength=3000* for sample KAN-C06235, and *minReadLength=4000* for sample KAN-130495. All assemblies were assessed for quality using the CheckM lineage workflow (v1.1.2) [18] and the average nucleotide identity (ANI) was calculated for all genome pairs using fastANI (v1.31) [19] with the option ‘many-to-many’ and default parameters. We excluded assemblies with deviating genome number of contigs >1000, less than 95 % pairwise ANI with any of the MKC reference genomes (*M. kansasii* ATCC12478, CP006835; *M. persicum* AFPC-000227, MVIF01; *M. pseudokansasii* MK142, UPHU01; *M. ostraviense* 241/15, NKRE01; *M. innocens* MK13, UPHQ01; *M. attenuatum* MK41, UPHT01; *M. gastri* DSM43505, LQOX01), and with more than 10 % of contamination as estimated by CheckM. The resulting data set contained 665 genomes (Table S2), in which 342 were generated in this study and 323 retrieved from public databases. Genomes were annotated with Prokka (v1.14.6) [20].

### Pangenome inference and core genome definition

The MKC pangenome was calculated and visualized using Roary (v3.13.0) [21] and Panaroo [22]. Since the MKC harbour seven species, we generated three pangenome runs for each pangenome software applying different sequence identity cutoffs (Roary parameter *-i* and Panaroo parameter *-c* set to 90 %, 80 %, and 70 %), to verify the impact in the pangenome estimation. The remaining parameters were the same for all runs, as follows: Roary parameters *-s* (no paralog splitting), *-cd 99* (a gene must be in 99 % of isolates to be core) and *-e --mafft -n* (core gene alignment generated with MAFFT) (v7.487) [23]; Panaroo parameters *--clean-mode strict --remove-invalid-genes --len_dif_percent 0.7*. Pangenome matrixes were visualized with Phandango [24] and accumulation curves with Panstripe [25].

### Phylogenomic analysis

For the MKC phylogenetic analyses, we generated a concatenated alignment of the core 1358 shared genes, that was created with Roary applying the following parameters: ‘*-i 95 s -cd 100 -e --mafft -n*’. Thus, a starting tree was built using RAxML-NG (v1.0.1) [26] applying a GTR model, Gamma correction and 100 bootstrap replicates. The same genome alignment was fed into fastGEAR (v1.0.1) [27] to detect recombinant regions. These regions were then parsed into a text file storing recombinant genomic regions as coordinates (as a BED file format – Browser Extensible Data) to generate a recombination masked alignment using maskrc-svg (first release, https://github.com/kwongj/maskrc-svg) and the starting tree. Hence, the masked alignment was used to build a recombination-free maximum-likelihood (ML) phylogeny in RAxML-NG with the same approach as above plus ascertainment bias correction, using the Lewis’ method (parameter +ASC_LEWIS). The final tree was employed and annotated in iTOL (v5) [28]. The core gene alignment length encompassed 334 144 sites, amounting to 222 205 760 characters for the whole data set. Across all isolates, 85 633 543 positions (38.53 % of all core gene alignment sites) were masked for recombination. For the final recombination-free ML phylogeny analysis, all invariant sites were removed with snp-sites (v2.5.1) [29] to obtain the final alignment length of 48 820 variants.

The *M. kansasii* species (former subtype I), the *M. kansasii* lineage L1.1 (460 genomes with available DNA sequencing reads, Table S12), and the *M. kansasii* Czech isolates phylogenies were inferred based on genome alignments generated with Snippy (v4.6.0, https://github.com/tseemann/snippy). Alignments were analysed with Gubbins (v2.4.1) [30] to detect and mask recombinant regions. After, invariant sites were removed with snp-sites and the resultant alignments were then used to detect phylogenetic lineages or sub-lineages and to generate a ML phylogenies with RAxML-NG, applying a GTR model, Gamma correction, Lewis’ method ascertainment bias correction and 100 bootstrap replicates. Lineages and sub-lineages were detected by applying a hierarchical Bayesian Analysis of Population Structure (hierBAPS) implemented in R (v1.0.1) [31] with a maximum depth of two and 50 as a maximum population number. Additionally, the Gubbins output analysis of the *M. kansasii* lineage L1.1 was used as input to infer a dating phylogenetic tree with a Bayesian approach, by using BactDating (v1.1) [32] with 10^6^ iterations. The *M. kansasii* lineage L1.1 dated tree fed PastML to the ACR inference for lineage and geographical origin, applying the maximum likelihood prediction method MPPA (marginal posterior probabilities approximation) and evolutionary model F81. The pairwise SNP distance for *M. kansasii* Czech isolates was evaluated using snp-dists (https://github.com/tseemann/snp-dists). *M. kansasii* Czech clusters were defined using the library ‘cluster’ with R statistical programming language and applying threshold of ten SNPs.

### Detection of mobile genetic elements

The identification of contigs with a possible plasmid origin was performed by using Platon (v1.6) [33], viralVerify (v1.1, https://github.com/ablab/viralVerify), and searches with BLASTn [34,35]. For the BLASTn approach, we compared MKC contigs against a customized database with 36 425 prokaryotic reference plasmids available on the RefSeq database (release 207) together with complete plasmid sequences of three MKC genomes (strains MK142, KAN-960446, and KAN-C06235). We applied the following parameters: e-value 1e^−05^, 80 % of identity, and word size=11. We considered a contig as originated from plasmid as follows: (i) if a contig was classified as plasmid by both Platon and viralVerify; or (ii) if a contig was classified as plasmid only by one software (Platon or viralVerify) and had a BLASTn hit with a reference plasmid; or (iii) if Platon classified the contig as plasmid due to a hit with a mobilization protein profile and viralVerify resulted in ‘uncertain’, regardless without a BLASTn hit, as Platon is based on high-quality Hidden-Markov Model (HMM) profiles of relaxase protein families available on the MOBscan database [36]. To avoid false positives, we discarded contigs with a sequence size less than 5 kb and BLASTn hits with less than 50 % of coverage. Besides the Platon prediction of hits with reference replication, mobilization, and conjugation proteins in the predicted plasmids, we additionally sought for the occurrence of marker proteins of the reference plasmid pMK12478 (NC_022654). This survey was performed by extracting from viralVerify results hits with type VII (T7SS) and type IV (T4SS) secretion systems proteins, based on HMM profiles of protein families (Pfam) listed in Table S17.

Prophages were detected with VirSorter2 (v2.2.3) [37] and CheckV (v0.8.1, database v1.0) [38]. First, genome sequences were surveyed with VirSorter2 applying the parameters *--include-groups dsDNAphage*, *--min-length 5000,* and *--min-score 0.75*. After, we checked the VirSorter2 resultant fasta files (*final-viral-combined.fa*) with the *end_to_end* workflow of CheckV. Finally, the *proviruses* and *viruses* fasta files resultant from the CheckV analysis were submitted over again to VirSorter2, with the same parameters of the first step analysis. Sequences of the inferred prophages were annotated with DRAM-v (v1.2.4) [39]. Thus, to identify possible prophages species specific as well the similarity between MKC prophages and known mycobacteriophages, a bipartite network and clusters of the prophages and their gene content (putative encoded proteins) were generated with AccNET (v1.2) [40], employing the parameters *--threshold 1.12* and *--kp ′-s 1.8 -e 1e-5 -c 0.6′*. For the clustering and network analysis performed by AccNET we also included 161 representatives mycobacteriophages retrieved from PhagesDB [41] (http://phagesdb.org, accessed in August 2021 – Table S18), that encompass all mycobacteriophage clusters available on the PhagesDB. The cut point 0.90 was applied for clustering heights, as this quantile was able to define almost exactly the clusters available on the PhagesDB, and the resultant network was employed in Cytoscape (v3.7.2) [42].

### Resistome and virulome analysis

The survey of genes evolved with possible antibiotic resistance activity was based on Hidden Markov Models profiles of the ResFams database [43], using HMMER (v3.3.2) [44] and applying an e-value 1e^−09^.

Genes with putative contribution to virulence activity were surveyed in MKC genomes with Abricate (v1.01, https://github.com/tseemann/abricate), based on a custom database with 287 *M*. *kansasii* genes with recognized orthologs in *M. tuberculosis* H37Rv that encode for potential virulence factors found by Mussi *et al*. [5]. We applied a cutoff of 85 % identity and coverage.

### Antiviral defense systems identification

We define the presence of antiviral defence systems based on PADLOC (v1.0.3, database v1.2.0) [45] and DefenseFinder (first release) [46,47]. Firstly, systems were assigned within MKC genomes applying the PADLOC results and the defence systems BREX, Dnd, Paris, and RM were additionally inferred by DefenseFinder. Additionally, we sought for CRISPRs regions on the *M. kansasii* ATCC 12478 genome with CRISPRfinder online tool (https://crispr.i2bc.paris-saclay.fr/Server/, database version 2017-05-09) [48], and regions identified as ‘confirmed CRISPRs’ were blasted against all MKC genomes analysed in the work.

### Structural variants within *M. kansasii* species

We investigated regions of difference (RDs) within genomes of the *M. kansasii* species by adapting RDscan (first release) [49] to map DNA sequencing read data of the *M. kansasii* isolates against the *M. kansasii* ATCC12478 chromosome (NCBI version NC_022663.1). Once RDscan is based on a read mapping approach, we generated fake reads for samples obtained from NCBI at genome level, without publicly available DNA sequencing reads. The following RDscan analysis parameters were applied: *threshold: 0.05, DHFFC: 0.1, minSVLEN: 200,* and *maxSVLEN: 100 000*. Fake reads were generated with readSimulator (first release, https://github.com/wanyuac/readSimulator), applying Wgsim (first release) [50] to generate synthetic paired-end short reads under the parameters: *--iterations 10 --readlen 200 --depth 100 --opts '-e 0 r 0 R 0 X 0 h -S 5'*.

### Phage typing

Susceptibility to 14 phage types of the MKC strains at the RIVM (National Institute for Public Health and the Environment, Bilthoven, The Netherlands) was determined as described by Engel and Berwald [51].

## Results

### Global distribution and phylogeny of the *Mycobacterium kansasii* complex

**Fig. 1.**
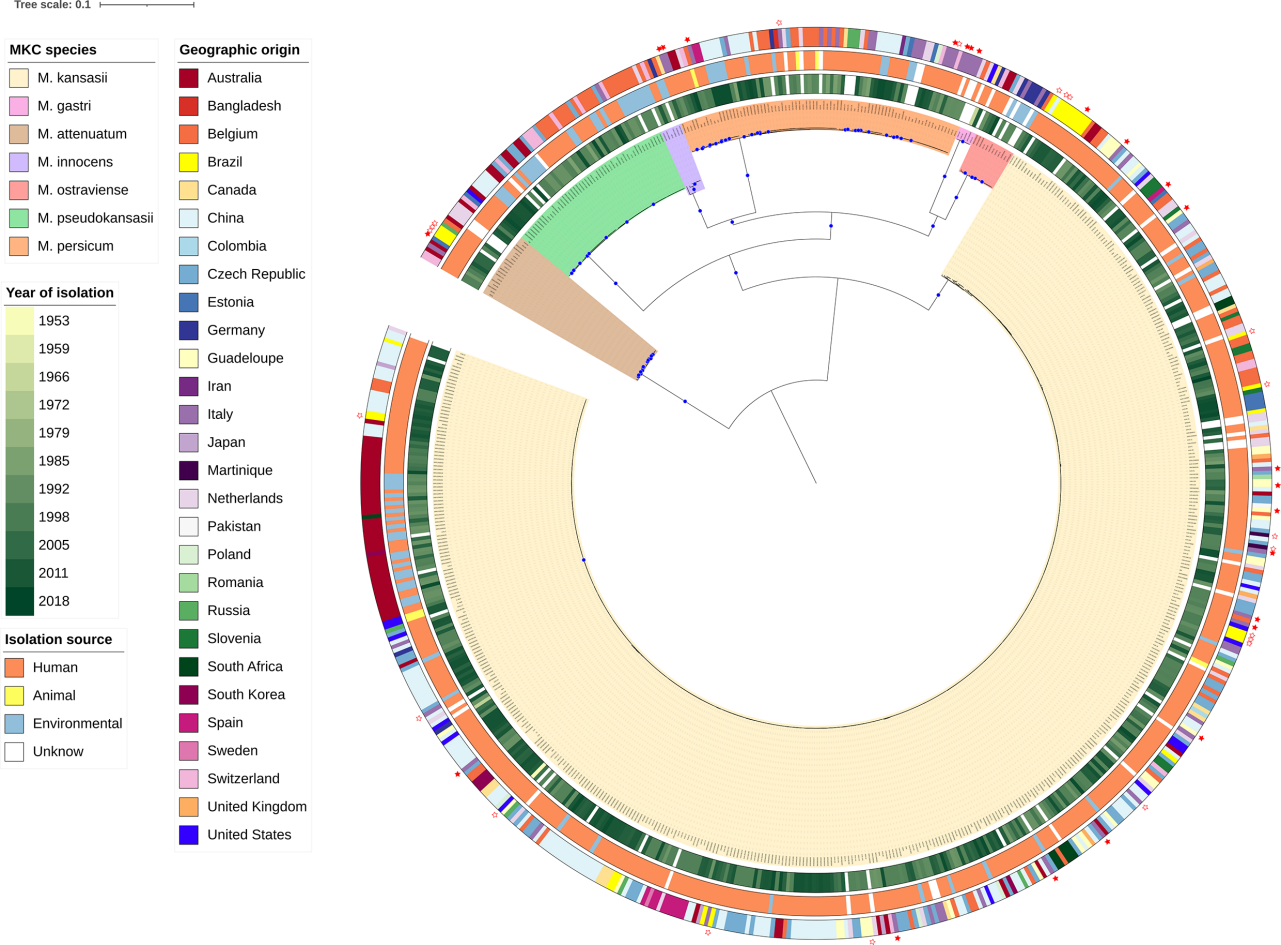
Midpoint rooted maximum likelihood phylogenetic tree of 665 *M*. *kansasii* complex genomes. Blue dots indicate nodes with bootstrap support above 75 % with shading of branches and nodes indicating the seven MKC species. The rings, from inside towards outside, designate: (**i**) the year of isolation (if available), (ii) the isolation source of the strains classified as either environmental, animal, human or unknown; (iii) the country of isolation. The outer red stars indicate clinical isolates with patient HIV information, being positive (solid) or negative (open).

To investigate the global distribution and genomic repertoire of the *M. kansasii* complex, we gathered whole genome sequencing (WGS) data of 722 strains, of which 388 were collected and sequenced in this study and 334 were obtained from public repositories with assembled genomes or available DNA short sequencing data (Table S1). After applying quality thresholds, ten samples collected from public repositories and 46 sequenced in-house were excluded (see Methods and Table S1). The final dataset analysed yielded 665 genome assemblies of strains from 28 countries and five continents remained for genomic and phylogenomic analyses. The isolation source distribution of the genomes is as follows: 538 isolated from humans, 76 from the environment, six from animals and 45 of unknown origin. Information on the colony morphology was available for 142 isolates (112 smooth and 30 rough) and HIV testing results were provided for patients associated with 44 isolates, including 24 positive and 20 negative (Fig. 1 and Table S2).

The genome sizes ranged from 5.63 Mb to 6.90 Mb and GC content was between 65.59 and 66.38 %. From the data collected in this study, we performed long-read sequencing technology for three genomes, resulting in fully closed genomes for two samples (KAN-960446 and KAN-130495), each presenting two replicons (a plasmid and a chromosome sequences), and one nearly fully closed genome (sample KAN-C06235) that was composed by nine contigs, including a complete plasmid sequence.

### Prevalence and distribution of plasmids among MKC

By applying and combining three *in silico* strategies, we were able to identify 346 contigs classified as putative plasmids (Table S3) within 244 MKC genomes (~37 % of 665). Most of these genomes presented only a single contig classified as deriving from a plasmid (*n*=169; 69 %), while the others contained two to five contigs per genome classified as plasmid-derived. The length of the contigs predicted as plasmid-derived ranged from 5423 to 237 122 base pairs with a mean and median of 39.6 and 20.1 kb, respectively. Around 47 % (163/348) of the putative plasmids encoded one (*n*=162) or two (*n*=1) tRNA genes. Five MKC species had plasmid-derived contigs with the following distribution: *M. kansasii* (176/492, 35.7 %), *M. persicum* (21/81, 25.9 %)*, M. pseudokansasii* (33/50, 66 %)*, M. ostraviense* (1/12, 9 %)*,* and *M. attenuatum* (14/19, 73.7 %); *M. innocens* and *M. gastri* contained no predicted plasmids (*P* < 0.05, Fisher’s exact test).

Among the 346 plasmid-derived contigs, 283 had no similarity with reference plasmid sequences, whereas 63 were matched via BLASTn. From the hits returned, 51 were similar to described MKC plasmid sequences while 12 were similar to plasmid sequences from other *Mycobacterium* species (*M. intracellulare* subsp*. chimaera*, *n*=4; *M. kubicae*, *n*=2; *M. aubagnense*, *n*=6). Based on marker genes, at least one conjugative (122/244), mobilizable (161/244) or replicative (130/244) mechanism was identified in each of the 244 genomes with predicted plasmids (Table S3 and Fig. S1).

### Prophages diversity and characterization across MKC

**Fig. 2.**
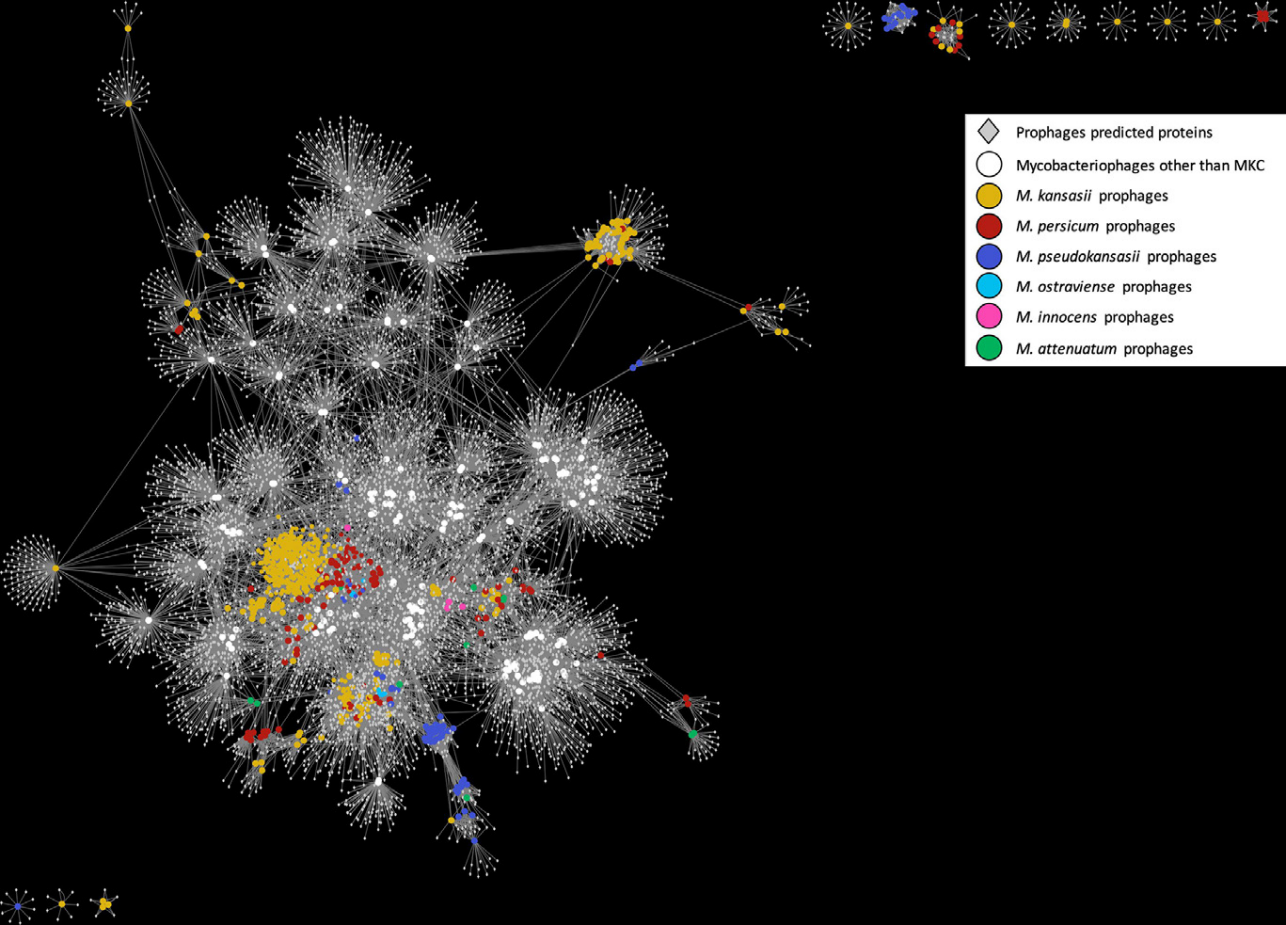
The bipartite network of the MKC prophages and their gene content. The predicted genes of the prophages are shown as grey diamonds, whereas prophages genomes as coloured circles. Genomes circles are coloured by MKC species or mycobacteriophages genomes obtained from hosts other than MKC species. Genomes are connected to their predicted genes by edges.

A total of 786 prophages were predicted to be distributed across 527 MKC genomes (~79.3 % of 665) (Table S4), with one to six prophages per genome. Prophage lengths ranged from 4.5 Kb to 100.2 Kb, and 109 prophages encoded one (*n*=70), two (*n*=6), four (*n*=32) or 21 (*n*=1) tRNA genes. Considering all MKC species, prophages were not detected in * M. gastri,* while prophage distribution across the other MKC species was as follows: *M. kansasii* (399/492, 81.1 %), *M. persicum* (73/81, 91.1 %)*, M. pseudokansasii* (36/50, 72 %)*, M. ostraviense* (9/12, 75 %)*, M. innocens* (3/6, 50 %)*,* and *M. attenuatum* (7/19, 36.9 %) (*P* < 0.05, Fisher’s exact test). Although MKC prophages were grouped into 59 clusters, distinct from the 37 mycobacteriophages clusters found in the PhageDB, the employed network based on the comparison and clustering process of prophages sequences, as well as their predicted genes, revealed a relationship between most MKC prophages and other known mycobacteriophages, as evidenced by the edges connections indicating their shared genes (Fig. 2 and Table S5). Around 90 % of prophages (710/786) were grouped into 20 clusters with at least five similar prophages, and the biggest cluster encompassing 335 prophages specific to *M. kansasii* (Fig. S2). Regarding the relationship between prophage clusters and host-species, 42 clusters were species-specific (found in only one species), whereas 17 were diverse, being shared by two to five different species. No prophage cluster contained all MKC species.

### MKC phage types

Among 18 MKC strains, we had information on susceptibility to 11 phage types, as outlined in Table S19. Phage type was characterized for 14 isolates of *M. kansasii*, two being phage type 2, 3, 7, 9, 10 and 13 each; a single strain of *M. kansasii* being phage type 6 and 8. Two strains of *M. attenuatum* were types: one was phage type 1 and the other phage type 6. Finally, a strain of *M. ostraviense* and *M. persicum* were of phage type 4 and 14, respectively.

### The MKC pangenome is open and has a highly diverse and species-specific accessory genome

Due to the diverse nature of the complex, we investigated whether different identity cut-offs or pangenome pipeline could substantially impact the MKC pangenome prediction. Thus, we generated pangenomes with Roary and Panaroo applying 70 %, 80 and 90 % of sequence identity cut-offs. For all the six runs performed, the pangenome resulted in a large amount of accessory genes, suggesting a highly diverse accessory genome for the complex. The number of core genes in the MKC pangenome was very similar and consistent for most runs, except for Roary with 90 %, where we observed a decrease in core genes (Panaroo: 70 %, 3648; 80 %, 3665; and 90 %, 3557. Roary: 70 %, 3659; 80 %, 3662; and 90 %, 3154). Notably, all Panaroo executions predicted almost the same total number of genes in MKC pangenome (70 %, 17 525; 80 %, 17 587; and 90 %, 17 750), reaching around 13 000 accessory genes. Instead, the Roary runs estimate a higher number of genes in the pangenomes (70 %, 18 741; 80 %, 21 104; and 90 %, 25 873). The pangenome accumulation curves using either software characterize an MKC opened pangenome, although those from Panaroo shows a discrete increase of new genes in its final portion (Fig. S3). Our results with Panaroo demonstrated greater robustness and, by comparing the pangenome matrices for each cut-off performed, it is clear that each MKC species is characterized by a distinct accessory gene content (Fig. S4).

### The MKC virulome is species specific

Among the 287 genes we detected that were encoding for potential virulence factors, 123 were present in all 665 samples, regardless of the species, while 164 genes were found between 39.9 % (265/665) and 99.9 % (664/665) of the samples (Table S6). The mean and median values of virulence factor count identified per MKC species was as follows: *M. kansasii* (281/283), *M. persicum* (259/260), *M. ostraviense* (251/252), *M. innocens* (247/247), *M. pseudokansasii* (246/248), *M. gastri* (244/244), *M. attenuatum* (229/229) (Fig. S5). A closer look at the 63 genes belonging to the type VII secretion system (T7SS) that has been described as playing a crucial role in mycobacterial physiology and virulence, showed that only 18 were found in all samples (Fig. S6). For VFs other than genes of the T7SS, 119 where not found in all samples (Fig. S7). Thus, for both VFs belonging or not to the T7SS system, we could identify that some genes were absent in some species different from * M. kansasii*, suggesting that these could be part of a specific virulome for each MKC species (Figs S6 and S7).

### The MKC antiviral arsenal distribution and diversity

We identified 17 distinct antiviral defence systems among the MKC species (Table S7). Wadjet, Restriction-Modification (RM) and Abortive infection (Abi) were the most abundant systems in MKC and were present in all MKC species, with frequencies of respectively 99.6 % (662/665), 96.4 % (641/665) and 83.2 % (558/665) of the genomes. However, while Wadjet and RM were present in most MKC species, Abi was uncommon in *M. attenuatum*, *M. innocens, M. ostraviense* and *M. persicum*. Additionally, in terms of frequency, four other systems (Sirtuin like, Thoeris, Cas, and BREX) were overrepresented in at least one MKC species compared to the others, whereas ten systems did not present specificity to any species (Fig. S8). On average, MKC species encode four antiviral systems (3.95), with the average number per species varying from 3.30 to 5.83. The number of antiviral systems per genome varies widely from a minimum of one (as observed in three *M. attenuatum* genomes) to a maximum of eight.

In this study, we also identified 13 CRISPRs regions in the chromosome sequence of *M. kansasii* ATCC 12478; three of those were classified as ‘confirmed’ and ten as ‘questionable’ (Table S8). The first confirmed region is located between the genomic coordinates 4 428 523 and 4 428 729 and consists of a 26 bp direct repeat (DR) consensus and three spacers. This region seems to be specific to *M. kansasii* as these three spacers were present in all *M. kansasii* genomes and absent in the genomes from other MKC species. The second confirmed CRISPR is the biggest of about 3.3 kbp, starting at 4 890 197 and ending at 4 893 369 genomic coordinates, harbouring a 26 bp DR and 43 spacers. Spacers of this region were partially or totally shared/absent in MKC species other than *M. kansasii*, possibly representing taxonomic markers. Specifically for *M. kansasii* species, 16 spacers of this CRISPR region were present in all *M. kansasii* genomes, whereas the other 27 were detected in between 3 and 96 % of the *M. kansasii* genomes. The third and last confirmed region is placed between genomic coordinates 6 379 425 and 6 380 393, comprehends a 24 bp DR and 13 spacers. Spacers of this region were found to be totally present or absent in MKC species other than *M. kansasii*, alike the biggest CRISPR locus, and are massively present in *M. kansasii* genomes (≥98 %) (Fig. S9).

### The MKC resistome is species-specific and consists of a wide group of resistance mechanisms

**Fig. 3.**
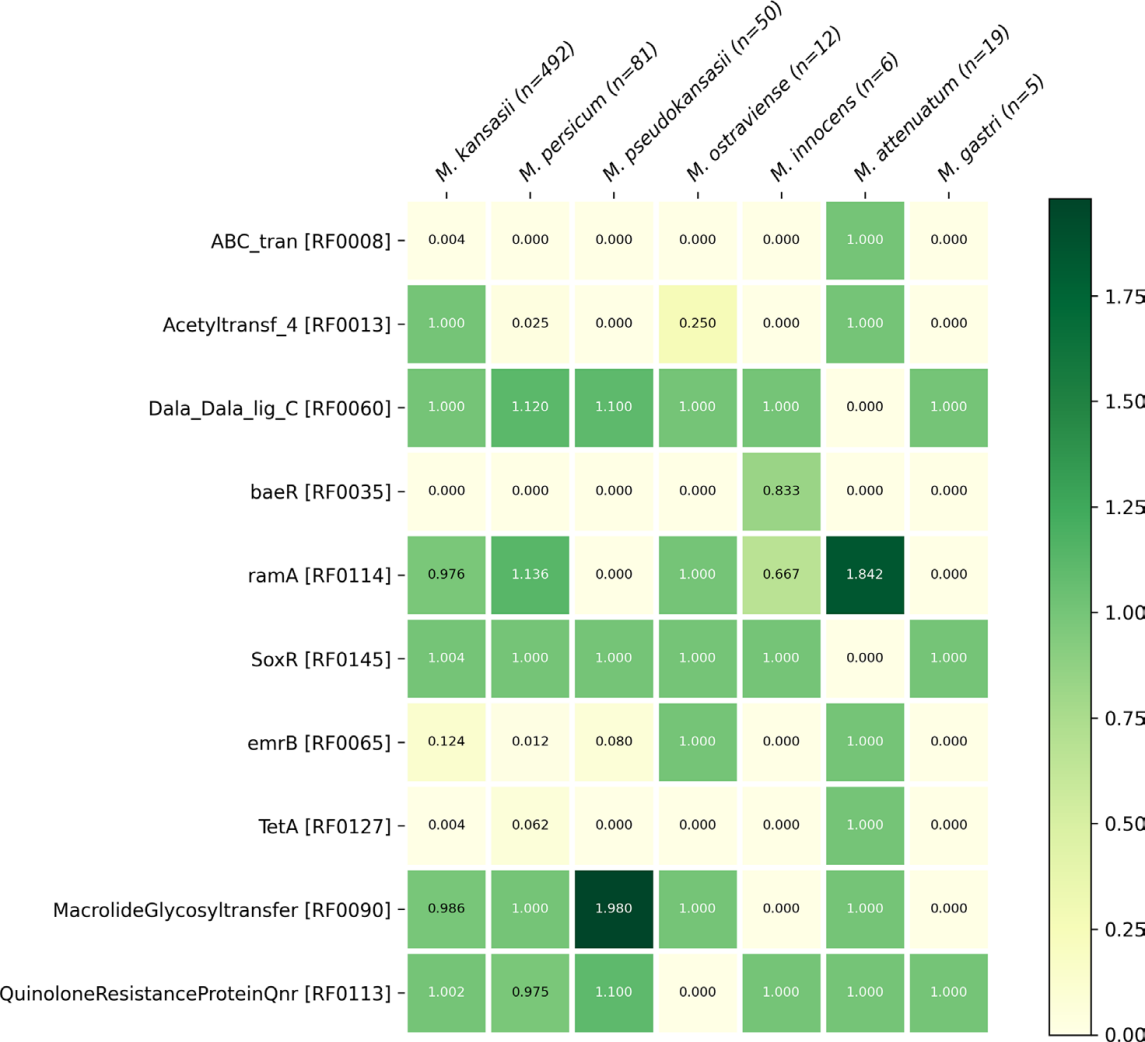
Resfams belonging to MKC species-specific. Heatmap showing the presence or absence of Resfams profiles (*n*=10) species related. Numbers inside cells indicate the average copy number of resistance protein profiles found in each group of MKC species genomes.

Looking for antimicrobial resistance protein profiles in the chromosome of MKC, we detected 83 resistance protein profiles (Resfams) belonging to 21 resistance mechanisms (Table S9 and Fig. S10). Since the database surveyed encompassed 173 Resfams related to 22 antimicrobial resistance mechanisms, the MKC resistome is extensive. We observed 57 profiles shared by all MKC species, but some of these profiles have different copy numbers per genome or even average copy numbers per species. Another ten Resfams profiles are apparently species-related as being absent in at least one MKC species (Fig. 3) and 16 Resfams were only occasionally observed in some genomes (Table S10). Thus, the presence and absence, as well as the variability number of copies, of Resfams within MKC characterize a species-specific resistome.

By comparing the MKC resistome with the Resfams found in the *M. tuberculosis* H37Rv (*n*=54), we observe two resistance mechanisms, comprising five Resfams, that are exclusives to MKC (Adenylyltransferase and RND Antibiotic Efflux). Nucleotidyltransferase was the unique resistance mechanism without representatives in MKC species and *M. tuberculosis* H37Rv whereas the remaining 19 resistance mechanisms were present in both. The Resfams belonging to those two resistance mechanisms MKC-exclusive were scarcely found in some genomes of the MKC (Table S10). Notably, all 54 Resfams found in the *M. tuberculosis* H37Rv are ubiquitous in MKC species, but many of them with distinct copy numbers. In the opposite way, some Resfams not found in *M. tuberculosis* H37Rv are also absent from some MKC species (Fig. S10).

We also investigated the resistome encoded by putative MKC plasmids and observed the presence of 27 Resfams related to 11 antimicrobial resistance mechanisms (Table S11). Interestingly, a Methyltransferase (RF0095 – Methyltransf_18) was the most commonly observed Resfam in plasmids, the profile also most frequent in chromosomes. However, whereas chromosomes carry several copies of this Methyltransferase, with the average number of copies per species ranging from 39.5 to 48.8, almost all predicted plasmids presented a single copy of this Methyltransferase, except for two *M. kansasii* isolates (KAN-130495 and SRR3666016), having two copies in their plasmid sequences.

### Distribution and emergence of *Mycobacterium kansasii* lineages and sub-lineages

**Fig. 4.**
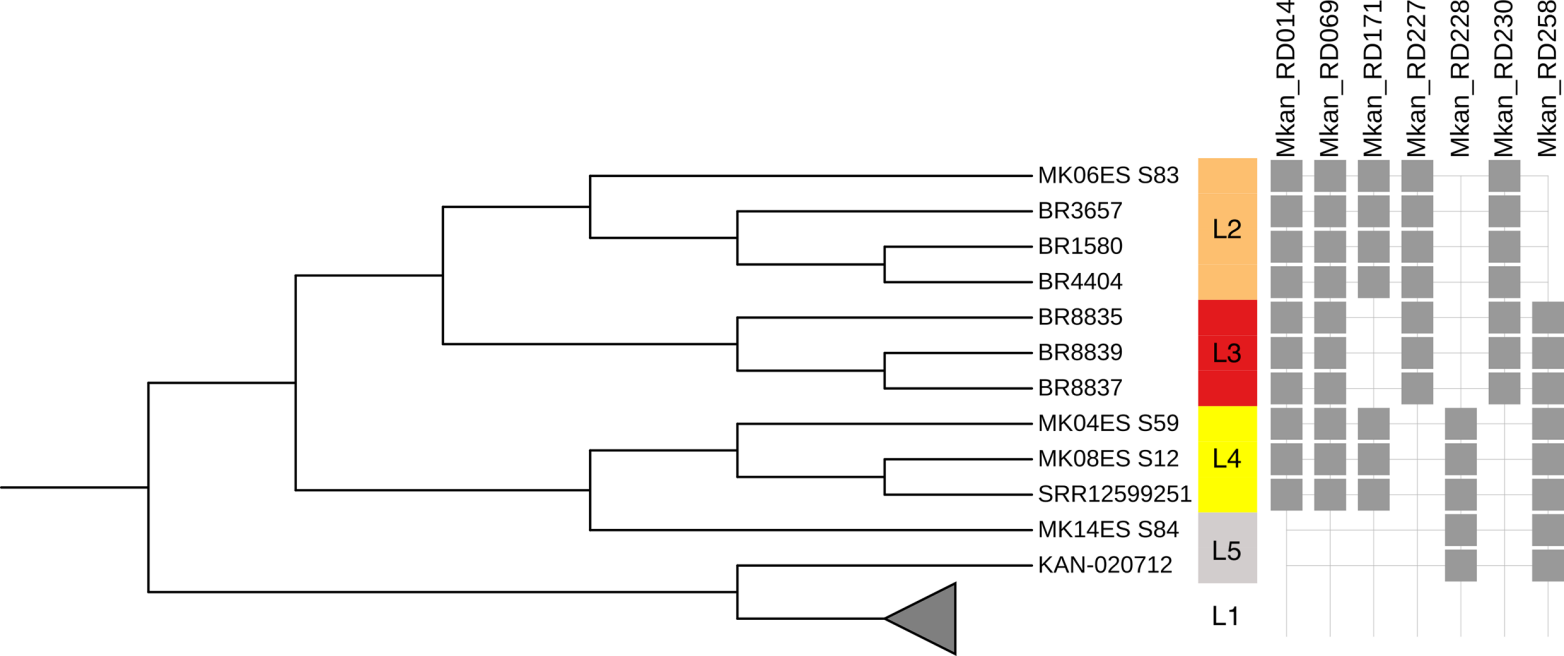
Regions of difference *M. kansasii* lineage specific. Maximum-likelihood phylogeny tree of 492 *M*. *kansasii* genomes, inferred based on nonrecombinant core SNPs, shows the *M. kansasii* genetic groups defined by BAPS and their specific RDs. Four genetic groups (L1–L4) characterize monophyletic lineages and two genomes (MK14ES_S84 and KAN-020712) belonging to L5 genetic group are unclassified due to their polyphyletic origin. Genomes belonging to the lineage L1 (*n*=480) are collapsed in the tree. Seven regions of difference specific to four (L2–L5) small represented genetic groups are indicated in grey sticks. The RD227 and RD228 shared a genomic region of 4 922 bp (genomic coordinates: RD227 from 4 766 718 to 4 771 663; RD228 from 4 766 741 to 4 787 038), even though RDscan defined as distinct RDs.

Using Bayesian analysis of population structure (BAPS) to analyse the *M. kansasii* (*sensu stricto*) species population distribution, five distinct genetic groups were defined among the 492 *M*. *kansasii* genomes. One major group, namely L1, comprises 480 genomes while the other 12 genomes were classified into four small genetic groups (L2, *n*=4; L3, *n*=3; L4, *n*=3; L5, *n*=2) (Table S12). By comparing the genetic groups defined by BAPS with the *M. kansasii* species phylogeny (Fig. 4), the groups L1, L2, L3 and L4 characterize four monophyletic lineages comprehending 490 genomes. Two genomes (MK14ES_S84 and KAN-020712) belonging to the L5 BAPS group were divided into distinct branches of the tree, with polyphyletic origin, thus needing further investigation of their evolutionary history.

Interestingly, from the 12 genomes belonging to L2-L5 BAPS groups, ten were from Brazil, one was from China (SRR12599251, L4) and one from Belgium (KAN-020712, L3). The main group L1 was further divided into three genetic groups, namely as L1.1, L1.2, and L1.3, with L1.1 as a dominant and comprehending 476 genomes, L1.2 with three genomes from South Korea and L1.3 with one genome from Belgium.

**Fig. 5.**
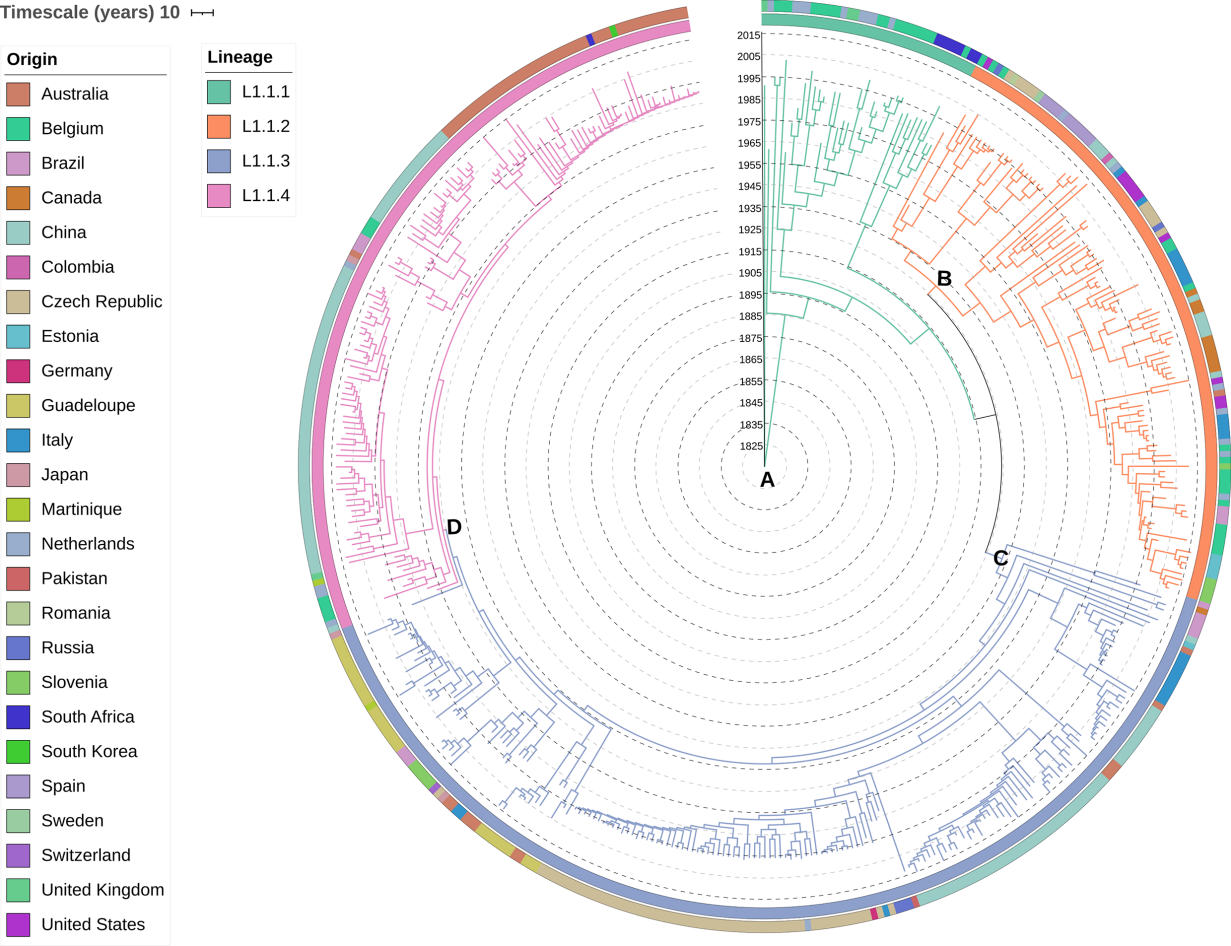
Maximum likelihood dated phylogeny of *M. kansasii* genomes (*n*=460) belonging to four sub-lineages. The outer circle indicates the country of origin of the isolate, whereas the first inner circle is the sub-lineage predicted in the Bayesian population analysis. The tree branches are also coloured accordingly to sub-lineages, showing the ancestral scenario reconstructed by PastML. The date of the most likely root was 1815, belonging to the older sub-lineage (L1.1.1, *n*=37) (**a**), with an unknown geographic region. Three recent sub-lineages emerged in (**b**) the United States in 1929 (L1.1.2, *n*=104), (**c**) Brazil in 1932 (L1.1.3, *n*=186), and (**d**) Australia in 1966 (L1.1.4, *n*=133) (the full tree visualization is also available at iTOL: https://itol.embl.de/tree/179702462428511716343991).

A further analysis of 460 genomes belonging to the main genetic group L1.1 with BAPS defined four L1.1 successful clonal clusters (namely as sub-lineages L1.1.1, *n*=37; L1.1.2, *n*=104; L1.1.3, *n*=186; L1.1.4, *n*=133). The Bayesian ancestral reconstruction estimated 1815 as most likely date of origin of the root of this main lineage with an evolutionary rate of 4.14e^−02^ (*P*<1.00e^−04^). An ancestral character reconstruction (Fig. 5) estimated the root of the four sub-lineages belonging to L1.1.1, with an unknown geographical origin as there were eight possible countries to root (Belgium, Brazil, China, Czech Republic, Italy, Netherlands, United Kingdom, or United States). The emergence of the other three sub-lineages was estimated as follows: L1.1.2, which include the reference strain ATCC12478, originated from United States in 1929 (marginal probabilities 0.99) and emerging from L1.1.1; L1.1.3 originated from Brazil in 1932 (marginal probabilities 0.82 and 0.99) and also emerging from L1.1.1; and L1.1.4 as the newest sub-lineage, arising in 1966 from Australia (marginal probabilities 0.86 and 0.99) and emerging from L1.1.3.

### Fixed mutations in *Mycobacterium kansasii* species could evolve impact on virulence and antimicrobial resistance

We analysed mutations specific or shared within *M. kansasii* sub-lineages (Table S13). Nine mutations were found to be almost exclusive to the older sub-lineage L1.1.1, five mutations shared by the recent sub-lineages L1.1.3 and L1.1.4, and two mutations shared by sub-lineages L1.1.1, L1.1.3 and L1.1.4 were almost totally absent in samples belonging to L1.1.2. Additionally, we observed that 24 positions of the reference strain ATCC12478 presented variants in more than 90 % of the *M. kansasii* genomes. Notably, 23 of these 24 mutations did not occur in a *M. kansasii* genome isolated in 1966 (6MK, CP_019885.1), suggesting that the reference positions are not part of the original *M. kansasii* wild-type or that these have been fixed more recently in the majority of the *M. kansasii* population (Table S14). Among these 24 variants, four occurred in genes (*cinA*, *embB*, *rpsL* and *rnj*) potentially associated with drug resistance in tuberculosis (TB) and three in genes (*dlaT*, *eccB* and *mycP*) required for virulence activity.

### Regions of difference of *Mycobacterium kansasii* and their possible contribution to the species diversity

In total, 14 621 occurrences of regions of difference (RDs) were observed in the data set of 492 *M*. *kansasii* isolates (Table S15) and were associated to 297 RDs defined by RDscan, with lengths ranging from 205 to 66 689 bp, and occurring in a range from a single to 442 genomes (Fig. S11). The three most frequent RDs occurred in over 80 % of the genomes and corresponded to two regions with transposases and one region encoding a PE family protein. The largest RD region, corresponding to 66 689 bp, was restricted to five genomes from Guadeloupe (KAN-117, KAN-119, KAN-160, KAN-163, and KAN-165). We also observed that seven RDs occurred only in 12 genomes of four small genetic groups (L2, L3, L4, and L5) (Fig. 4). Specifically to RDs restricted to L2–L5, despite RDscan having defined the RD227 and RD228 as distinct RDs, we noticed a shared genomic region of 4 922 bp between these two RDs (genomic coordinates: RD227 from 4 766 718 to 4 771 663; RD228 from 4 766 741 to 4 787 038), causing the loss of four coding sequences (MKAN_RS20805: ‘RtcB family protein’; MKAN_RS29915: ‘AAA family ATPase’; MKAN_RS20830: ‘XRE family transcriptional regulator’; and MKAN_RS20835: ‘type II toxin-antitoxin system RelE/ParE family toxin’), one tRNA (MKAN_RS20815) and one pseudogene (MKAN_RS20840). Therefore, the shared region of RD227 and RD228 likely characterize the distinction between the large lineage L1 and the other genetic groups L2–L5. There was a distinction in the RDs average per genome for the whole dataset (29.78, SD = 20.29, median=25) when compared with the four defined for sub-lineages (L1.1.1 = 21.35, SD=13.09, median=18; L1.1.2 = 21.76, SD=16.62, median=17; L1.1.3 = 25.30, SD=20.28, median=16; L1.1.4 = 40.89, SD=18.05, median=43) (Fig. S12).

### Analysis of *Mycobacterium kansasii* from the Czech Republic indicates a higher transmission rate for the sub-lineage L1.1.3

**Fig. 6.**
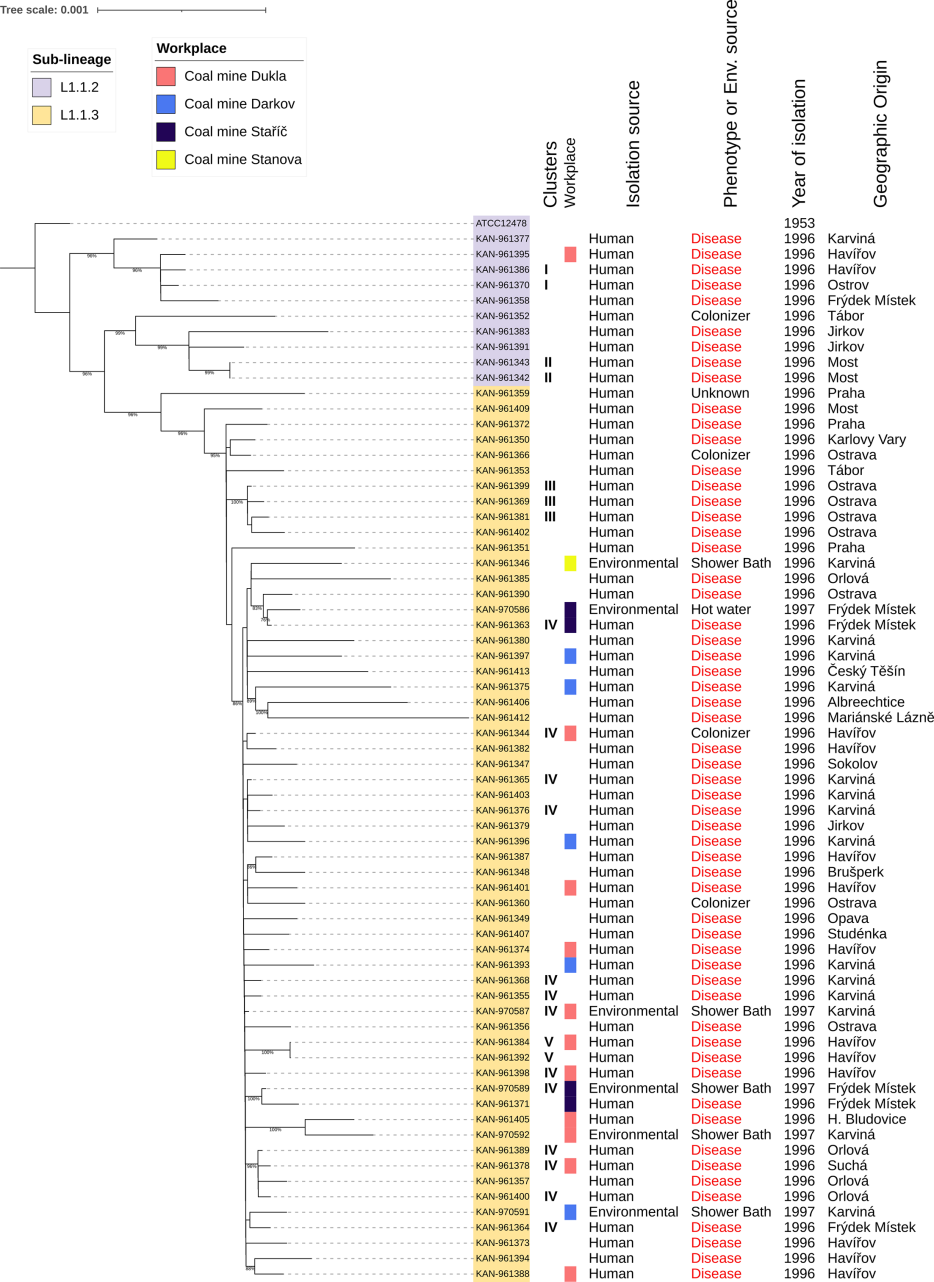
Maximum likelihood phylogeny tree of *M. kansasii* strains isolated in Czech Republic. The tree inferred based on nonrecombinant core SNPs is rooted in the reference strain ATCC12478. The number of nucleotide substitutions per site is indicated by the scale bar and bootstrap values are indicated in branches with at least 75 % of support. Sample sub-lineage is indicated by colours at node names. Beside the tree, from left to right, the following characteristics are described: Roman numerals indicating clusters of genomes with 0 to 10 SNPs pairwise distance; the workplace (where available); source of the isolation as either human or environmental; clinical phenotype (human isolates) or detailed isolation source (environmental isolates); year of isolation; and geographical origin of the isolate (environmental isolates) or patient (human isolates).

We further analysed a collection of 68 *M*. *kansasii* isolates from the Czech Republic collected in 1996 and 1997, in order to understand if some of them belonged to the same outbreak and looking for possible clusters of isolates with a pairwise genomic difference fewer than ten SNPs (Table S16). These isolates were either clinical (*n*=62) or environmental (*n*=6), the latter derived from artificial water sources in four distinct coal mines. Among the clinical samples, 15 were collected from employees of three of these mines. Additionally, from the 62 clinical isolates, 57 patients were considered with NTM disease, four colonisers, and one with unknown clinical phenotype. The genotyping showed that isolates belonged either to sub-lineages L1.1.2 (*n*=10) or L1.1.3 (*n*=58), suggesting they not belong to the same outbreak. The SNP distance between isolates ranged from 0 to 135, with a mean SNP distance between all Czech isolates was 41.55 (SD=26.78). Considering only isolates belonging to L1.1.2 sub-lineage the mean was 52.93 (SD=24.47), and 28.70 (SD=16.11) for isolates belonging to the L1.1.3 sub-lineage. The smaller distance for isolates from distinct lineages was 56, two pairs of isolates had zero SNP distance (KAN-961342 and KAN-961343, belonging to L1.1.2; and KAN-961384 and KAN-961392, belonging to L1.1.3). The pairwise distance between each Czech isolate with those whose belong to the same sub-lineage, the mean for L1.1.2 Czech isolates ranging from 41.22 to 71.66 whereas for L1.1.2 Czech isolates were between 15.05 and 68.03. Interesting, the sample with lowest mean distance (15.05) is an environmental sample (KAN-970587) isolated from a shower-bath in a coal mine. By applying the SNP distance thresholds of ten SNPs, five clusters were identified. Notably, one of these clusters include clinical, colonisers and environmental isolates (Fig. 6). Upon considering the geographic distribution of the lineages, we observed some genotypes were from isolates derived from patients of distant geographic origin.

## Discussion

The findings of this study highlight several genomic characteristics of the non-tuberculous mycobacterium *M. kansasii* complex, demonstrating that the seven known MKC species harbour unique and shared genomic signatures, more specifically in their pangenome, resistome, virulome, mobilome and defence systems. We expanded the publicly available MKC data by providing genome sequencing of 342 isolates and, in line with previous reports [2,3, 7], our data support the known MKC population distribution into seven distinct species. Additionally, we have provided new MKC WGS data for isolates from 28 countries, including ten countries with no MKC available data until now (Bangladesh, Colombia, Estonia, Guadeloupe, Martinique, Pakistan, Romania, Russia, Slovenia, and Sweden). After realizing our experiments, Zhang *et al*. [52] and Rajendran *et al*. [53] published data including MKC genomes from respectively 237 and 12 isolates from China and India.

Our results further illustrate that the MKC accessory genome is diverse, large, and species-specific. Additionally, this illustrates that mobile genetic elements contribute to the acquisition of new genes by MKC species, as documented by the high frequency of strains with plasmid and prophage signatures. Similarly to evaluated and showed to *Mycobacterium tuberculosis* complex pangenome estimation [54], we observed that the choice of pangenome pipeline considerably impacts pangenome size. Whereas Panaroo robustly estimated the MKC pangenome with similar size and characteristics, regardless of the similarity cut-off applied, the Roary overinflated the accessory genome size with an increased impact for higher cut-offs adopted.

The variability of virulence factors within MKC species is supported by earlier data, comparing *M. kansasii* against other species [5,9, 55] suggesting an MKC species-specific virulome. To address this, we defined 287 *M*. *kansasii* genes orthologs to * M. tuberculosis* H37Rv potential virulence factors and screened for their presence in the genomes collection. We observed unique and shared virulome characteristics among the MKC species with 123 VFs ubiquitous to MKC and 164 genes partially or totally absent from some MKC species or strains. In concordance with their frequent isolation from diseased humans, *M. kansasii* and * M. persicum* were the species with the highest number of virulence factors. Our virulome analysis also encompasses genes belonging to the type VII secretion systems (T7SS), which consists of five paralogous loci (ESX-1 to ESX-5) in *M. tuberculosis* and is known to play a crucial role in mycobacterial survival in the host, phagosome escape and immune response evasion [56]. Looking for orthologs of the *M. tuberculosis* H37Rv T7SS genes, the majority of *M. kansasii* isolates presented all orthologs as previously observed [8], while only two orthologs (*espG1* and *espI*) being absent in *M. persicum*. For the remaining species, the number of H37Rv T7SS orthologs not identified varies between six and 20. This again may explain the lower association of human disease and species other than *M. kansasii* and *M. persicum*.

Encoded outside the T7SS gene cluster, the *espACD* operon is recognized as essential for ESX-1 secretion [57]. In disagreement with a previous report [55], we observed that the presence of the *espACD* operon is not exclusive for *M. kansasii* and was also present in all *M. pseudokansasii* and *M. attenuatum* genomes. Considering that our data include several *M. persicum* isolates from clinical cases, this indicates that the *espACD* operon is not a unique feature for MKC species pathogenicity. Notably, one * M. kansasii* isolate (KAN-43) lacked the *espACD* operon, with a deletion of 6 874 bp that was also confirmed using RDscan analysis (Mkan_RD151). Still, with respect to the species or strains virulome characteristics, even though each MKC species generally keeps a specific set of VFs, some strains are deviating from their species virulome gene set, by losing some VFs. This poses that distinct strains of MKC species can present a broad virulence spectrum, as already observed for *M. kansasii* [5]. Interestingly, KAN-43 is one of the two strains that belonged to phage type 2 and if the absence of the *espACD* operon has influence on the strain’s virulence is currently under investigation.

We have proposed a holistic approach to address the MKC resistome by looking for resistance protein profiles (Resfams) across the analysed genomes. Similar to the MKC virulome, the MKC resistome is species-specific, and a high number of Resfams (*n*=83) within MKC species were identified, belonging to 21 of the 22 resistance mechanisms described. This suggests a large potential for resistance against xenobiotics and seems compatible with an organism lifestyle capable of growing in different environmental conditions. The MKC resistome is also larger than that of *M. tuberculosis* H37Rv and in general presents more copies of resistance profiles ubiquitous to both MKC species and H37Rv. The variability of Resfams copy numbers corroborates that distinct MKC species can have different antimicrobial phenotypes, specially related to intrinsic antimicrobial resistance levels. Our observation of the presence of 27 Resfams within MKC plasmids strengthens the hypothesis of expansion of the MKC resistome by lateral transfer events. DNA duplications constitute important precursors for genomic variation, and MKC species have a genome content greater than *M. tuberculosis*. Despite the current well-established knowledge of detecting SNPs in target genes, as occurring mostly in *M. tuberculosis*, the large accessory genome and strong recombination signal seen in MKC species suggests that acquisition of genes could be an additional mechanism for modifying the resistance phenotype to antimicrobials. As we have shown in the MKC resistome panorama and comparison to that of *M. tuberculosis* H37Rv, in general, MKC species harbour more Resfams copies than *M. tuberculosis* H37Rv. Treatment for MKC infections resembles the TB therapy scheme but seems challenging due to the longer period and demand for multiple drugs [58]. There is still little known about the impact of genomic differences between *M. tuberculosis* and MKC species that may result in distinct drug resistance mechanisms and more studies are needed and underway to define improvements to the current standard of care therapy for MKC infections, such as drug scheme and dosage.

We have identified putative plasmid-derived contigs within 36.7 % of the MKC genomes, slightly more than 29.3 % previously observed [6]. The larger frequency observed is probably due to our combined approach of three *in silico* strategies, as well as to the use of larger databases, not restricting the search to a few genetic markers and avoiding a selection bias, as such, expanding the knowledge on plasmids diversity within MKC genomes. All genomes with plasmids had at least one replicative, conjugative or mobilizable plasmid marker identified. As expected, homologs of the Type IV and Type VII secretion systems are common among MKC plasmids and besides their potential contribution to virulence, contribution to antimicrobial resistance was likely because of the presence of Resfams profiles.

Except for *M. gastri*, we found that prophages are common to MKC genomes. As the few known *M. gastri* strains with known origin come from humans, this species may be host-restricted, thus indicating a likely reason for the absence of prophages, due to limited exposure to phages and evolutionary adaptation. Notably, despite most MKC prophages and other known mycobacteriophages are related as evidenced by their shared genes connections, all predicted MKC prophage clusters were distinct from clusters of known phage representative sequences. Similar to the *M. abscessus* complex [59], our results suggest that phages can be exchanged by different MKC species, as many defined prophage clusters were shared by distinct MKC species. However, this does not exclude species phage affinity because some prophage clusters were species-specific. To what extent these prophages contribute to biological activity and are related to active phages needs further investigation.

As an extension to our analysis of presence of prophages, we identified the MKC arsenal of defence systems mechanisms and observed that most MKC genomes contain three distinct defence mechanisms. One is of the Wadjet type, conferring protection against plasmid transformation [60]; the second is the RM type, that acts by recognize and target sequences of bacteriophages [61]; and finally, the Abi type, an abortive system that, upon infection, leads towards cell death or metabolic arrest [62]. Besides these three, even though less frequent, another 14 defence systems were identified, showing the potential of different MKC species to acquire distinct anti-phage defence mechanisms. However, given the observed variability and diversity of defence mechanisms and the limited number isolated from some of the MKC species available, the acquisition and maintenance of such systems in MKC genomes must be further investigated. In addition, we identified three different CRISPR regions along the reference genome of the strain *M. kansasii* ATCC12478, including the identification of spacers with variable presence along strains and species of the complex. This points to possible differences in specificities of defence against phages and could lead to the development of a typing system such as spoligotyping used for the *M. tuberculosis* complex [63]. Interestingly, CAS defence systems were inferred in only 5 % of *M. kansasii* genomes and not detected in the ATCC12478 strain. So, these CRISPR regions are isolated from a *cas* loci and do not constitute a CRISPR-CAS system, as observed in other bacterial genomes [64].

Besides confirming the seven main species within the MKC in the largest dataset analysed so far, our findings demonstrate that the *M. kansasii* species can be subdivided into four monophyletic lineages, which was similar as previously observed [9,12]. One of these, the major lineage (L1), is globally distributed and comprises four successful clonal groups (sub-lineages L1.1.1, L1.1.2, L1.1.3 and L1.1.4). We observed that Brazil is an important source of *M. kansasii* diversity, as ten of 12 genomes from the underrepresented genetic groups L2–L5 had been isolated in the country. This genetic diversity of *M. kansasii* strains from Brazil has been previously suggested [12], but it is remarkable that from almost 500 strains this group of few diverse genomes is mostly restricted to Brazil. This urges the need to investigate more strains from this country for better understanding of the L1 local and global phylogeny. We evidenced that three (L1.1.2, L1.1.3 and L1.1.4) of the four most represented and successful sub-lineages emerged during the twentieth century, with the newest sub-lineage since the 1960s. The highest frequency of deletions was observed in strains of the most recent sub-lineage L1.1.4. Additionally, we observed that *M. kansasii* genetic groups L2–L5 are characterized by a region of difference (RD) of 4 922 bp, resulting in loss of one tRNA, one pseudogene and four coding sequences that include a RtcB protein, an ATPase, a XRE transcriptional regulator and a type II toxin-antitoxin RelE/ParE.

Regarding genetic differences among *M. kansasii* we observed SNPs found in all or almost all genomes analysed and some of these mutations were observed in genes with potential impact on virulence (*dlaT*, *eccB*, and *mycP*) and antimicrobial resistance (*CinA*, *embB, rpsL,* and *rnj*) [65,70].

Transmission methods of *M. kansasii* are not well elucidated and there are not defined thresholds of SNPs distance to infer transmission clusters. A suggestive acquisition of human *M. kansasii* infections from contaminated water was previously observed in a group of isolates with 0–7 SNPs distance [7]. Here, by considering an exploratory cut-off of ten SNPs in the collection of strains from Czech Republic, we observed colonisers, clinical and environmental isolates from distinct geographic region belonging to the same cluster. Environmental samples of this collection were from the water supply system of four coal mines, indicating a shared environmental source as a common route of *M. kansasii* infections, with an important role for contaminated water.

## supplementary material

10.1099/mgen.0.001266 Uncited Fig. S1.

10.1099/mgen.0.001266 Uncited Table S1.

## Abbreviations

Abi: abortive infection
ANI: average nucleotide identity
BAPS: bayesian analysis of population structure
BED: browser extensible data
CRISPR: clustered regularly interspaced short palindromic repeats
DR: direct repeat
HIV: human immunodeficiency virus
HMM: Hidden-Markov Model
MKC: *Mycobacterium kansasii* complex
ML: maximum-likelihood
MPPA: marginal posterior probabilities approximation
NGS: next-generation sequencing
NTM: non-tuberculous mycobacteria
Pfam: protein families
RD: region of difference
Resfams: resistance protein profiles
RM: restriction-modification
SNP: single nucleotide polymorphism
SRA: sequence read archive
TB: tuberculosis
T4SS: type IV secretion system
T7SS: type VII secretion system
VF: virulence factor
WGS: whole genome sequencing
