# Supplementary material for: Phylogenomic and genomic analysis reveals unique and shared genetic signatures of Mycobacterium kansasii complex species
Source: Microb Genom. 2024 Jul 17;10(7):001266. doi: 10.1099/mgen.0.001266 (PMC11316565; doi:10.1099/mgen.0.001266)
Supplement: Uncited Table S1. [file mgen-10-01266-s002.pdf]

Description of Additional Supplementary Files

File Name: Supplementary Data 1
Description: Supplementary tables 1 to 19 in excel format providing details of used data and results of this study, as follows:

Supplementary table 1: List of MKC WGS data collected and analyzed, including accession numbers and references.

Supplementary table 2: Associated metadata for all 665 MKC genomes analyzed and included in phylogenetic analysis.

Supplementary table 3: List of predicted plasmids per MKC genome and their characteristics.

Supplementary table 4: List of predicted prophages per MKC genome and their characteristics.

Supplementary table 5: Details of the prophages clusters.

Supplementary table 6: Detailed presence and absence of MKC genes orthologs to the 287 *M. tuberculosis* H37Rv genes coding putative virulence factors.

Supplementary table 7: Detailed presence and absence of Antiviral systems predicted in MKC genomes.

Supplementary table 8: List of confirmed and questionable CRISPRs in the *M. kansasii* ATCC 12478 genome and their characteristics.

Supplementary table 9: List of Resfams found in MKC genomes per species.

Supplementary table 10: List of 16 Resfams occasionally occurred in some MKC genomes.

Supplementary table 11: List of Resfams found in the MKC predicted plasmids.

Supplementary table 12: List of *M. kansasii* lineages and sublineages define by BAPS.

Supplementary table 13: List of mutations exclusive to specific *M. kansasii* sublineages.

Supplementary table 14: List of *M. kansasii* mutations found in at least 90% of the *M. kansasii* isolates.

Supplementary table 15: List of regions of difference (RDs) in *M. kansasii* genomes.

Supplementary table 16: Detailed metadata of *M. kansasii* isolates from Czech Republic.

Supplementary table 17: List of HMM profiles investigated in predicted plasmids.

Supplementary table 18: List of reference mycobacteriophages genomes included in the prophages clustering analysis.

Supplementary table 19: List of samples with phagetype analyzed.

File Name: Supplementary Data 2
Description: Supplementary Figures 1 to 5 and 8 to 11.

File Name: Supplementary Data 3
Description: Supplementary Figure 6.

File Name: Supplementary Data 4
Description: Supplementary Figure 7.
