# Supplementary material for: Phylogenomic and genomic analysis reveals unique and shared genetic signatures of Mycobacterium kansasii complex species
Source: Microb Genom. 2024 Jul 17;10(7):001266. doi: 10.1099/mgen.0.001266 (PMC11316565; doi:10.1099/mgen.0.001266)
Supplement: Uncited Fig. S1. [file mgen-10-01266-s001.pdf]

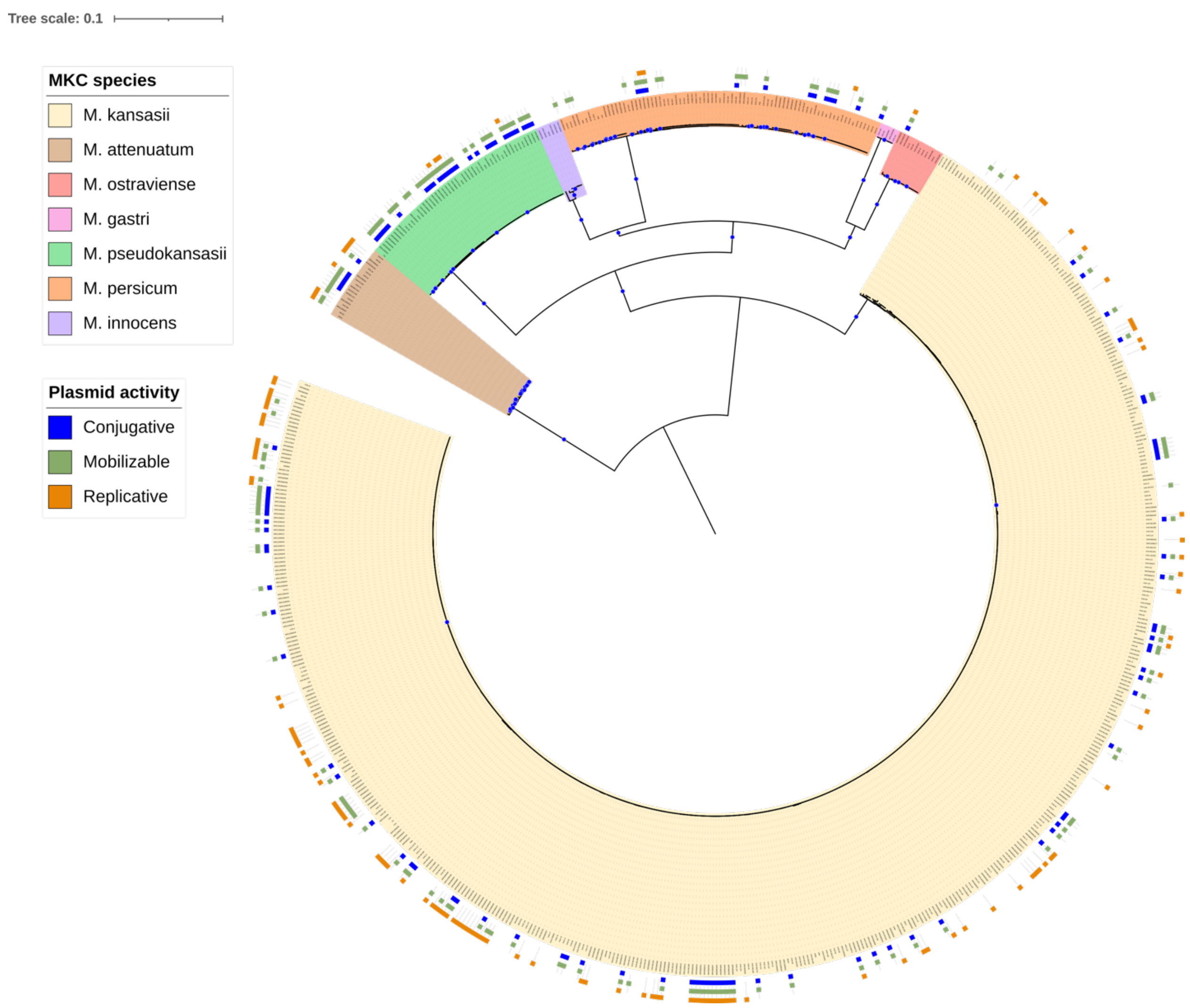

Supplementary Figure 1. **Plasmids distribution among MKC**. Midpoint rooted maximum likelihood phylogenetic tree showing the prevalence and distribution of plasmids among 665 *M. kansasii* complex genomes. Blue dots indicate nodes with bootstrap support above 75% with shading of branches and nodes indicating the seven MKC species. The three outer rings denote the plasmids machinery activity inferred, as either conjugative, mobilizable or replicative.

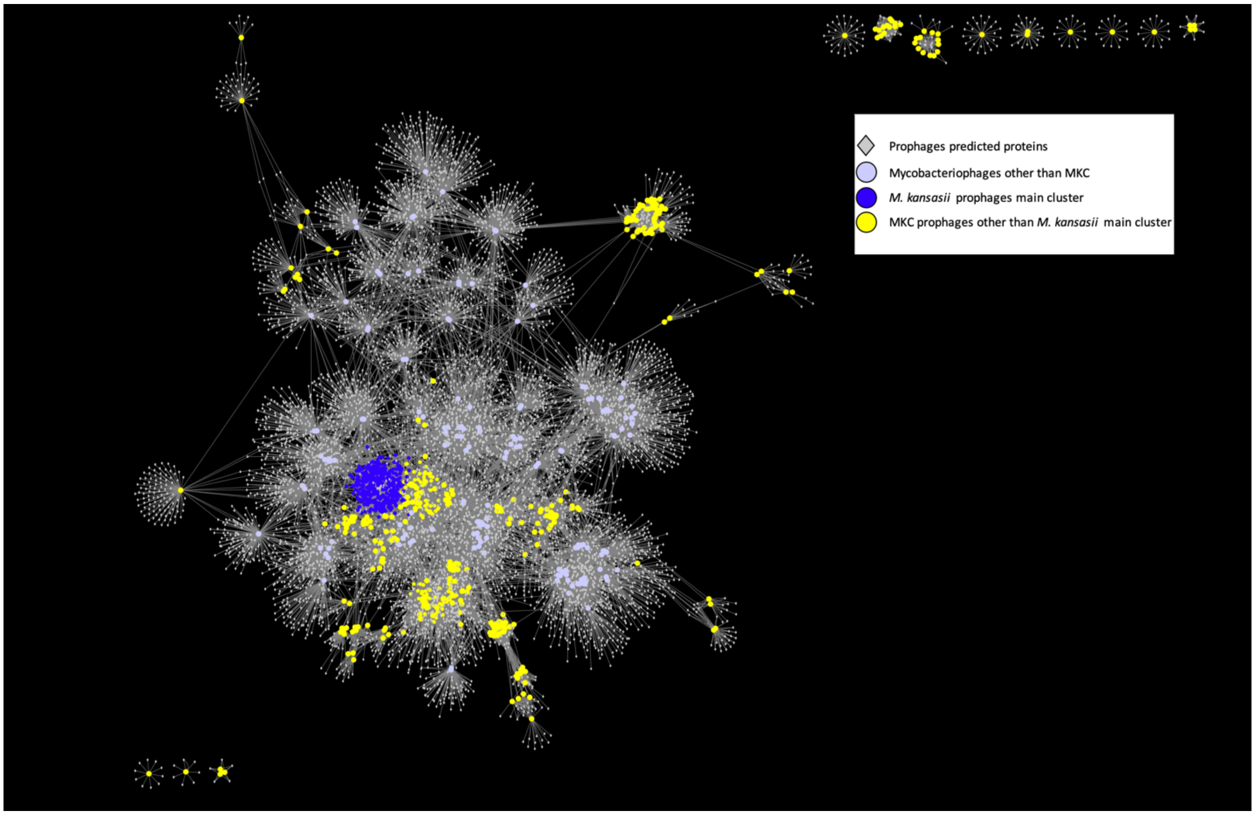

Supplementary Figure 2. **MKC prophages network**. The bipartite network of the prophages and their gene content. The predicted genes of the prophages are show as grey diamonds, whereas prophages genomes as colored circles. (A) Genomes circles are colored by MKC species or Mycobacteriophages genomes obtained from hosts other than MKC species, as defined in the right upper legend. Genomes are connected to their predicted genes by edges. (B) Genomes belonging to the main *M. kansasii* cluster, with 335 similar prophages, are show as dark blue circles. MKC prophages genomes belonging to clusters other than the main *M. kansasii* prophage cluster and Mycobacteriophages genomes are, respectively, represented by yellow circles and light blue circles.

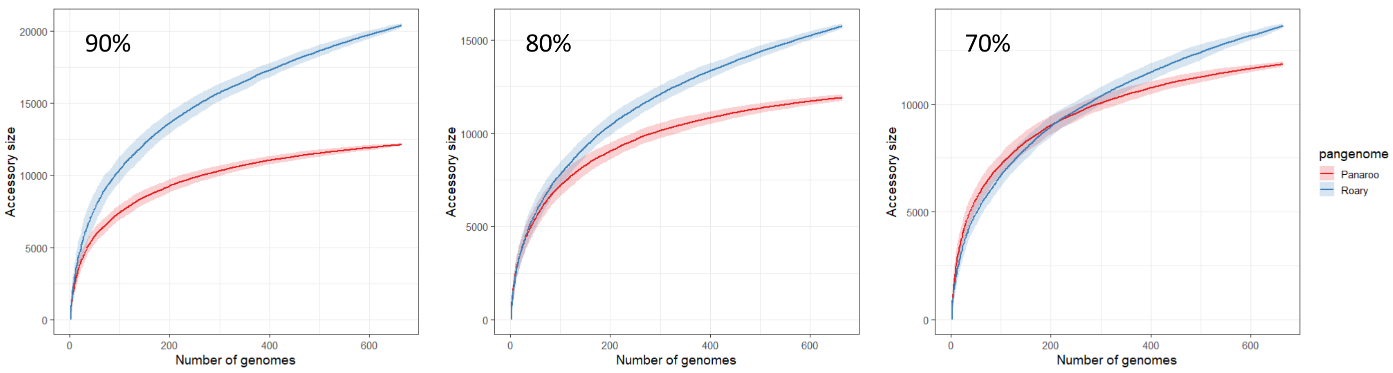

Supplementary Figure 3. **MKC pangenome accumulation curves** applying three different similarity cut-offs (90%, 80%, 70%). Each graph shows two curves representing a comparison between Panaroo and Roary results. The graphs axis represents the number of genes (vertical) accumulated in the MKC accessory genome *versus* the number of genomes (horizontal).

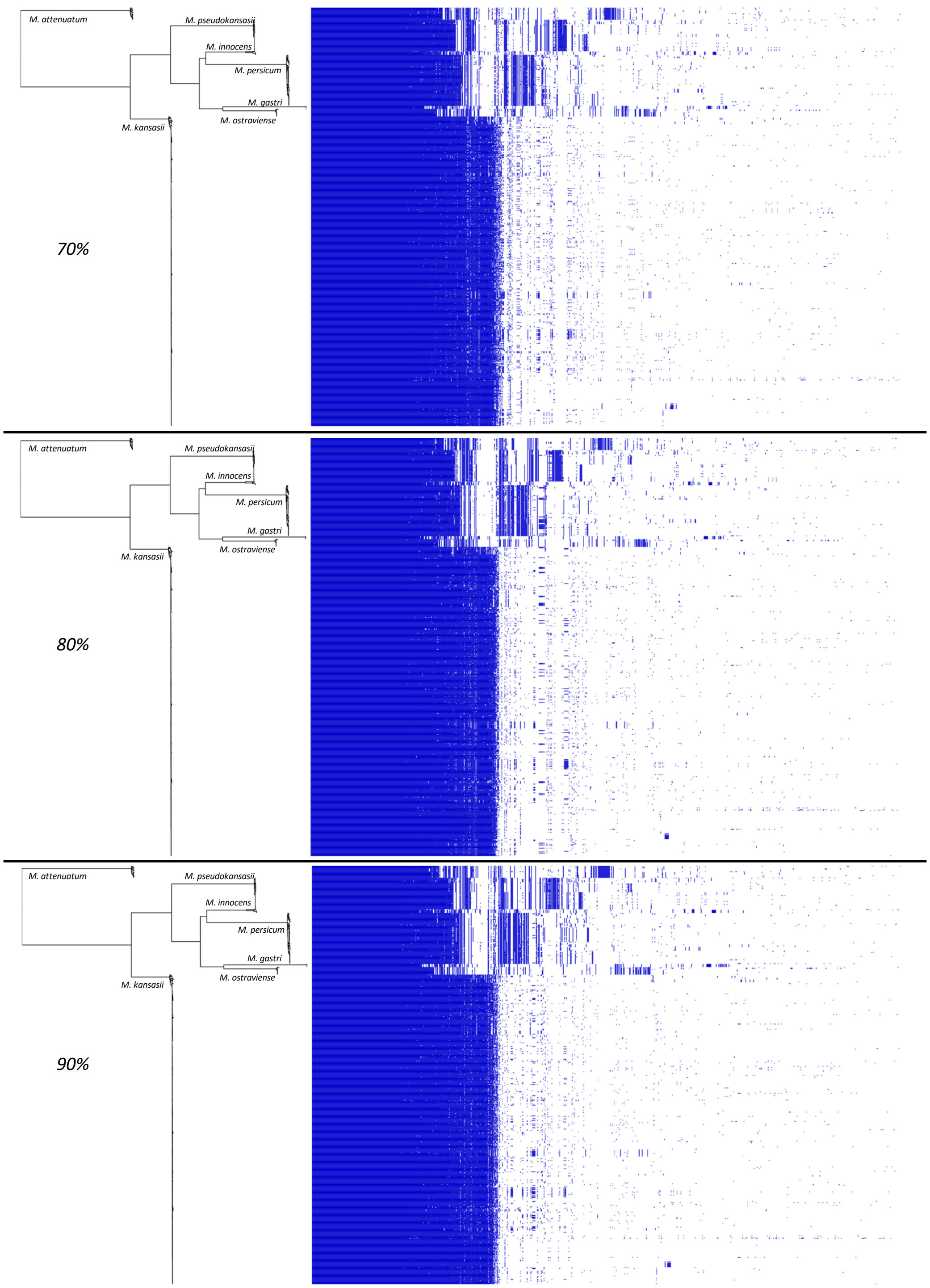

Supplementary Figure 4. **MKC pangenome matrix**. The *M. kansasii* complex accessory gene content is structured by species regardless the similarity cut-off applied (70%, 80% or 90%, from top to bottom). The pangenome matrixes shows the presence (blue) or absence (blank) of homologues genes among MKC species genomes are plotted (right), and the phylogenetic trees (left) corresponds to the tree in Figure 1.

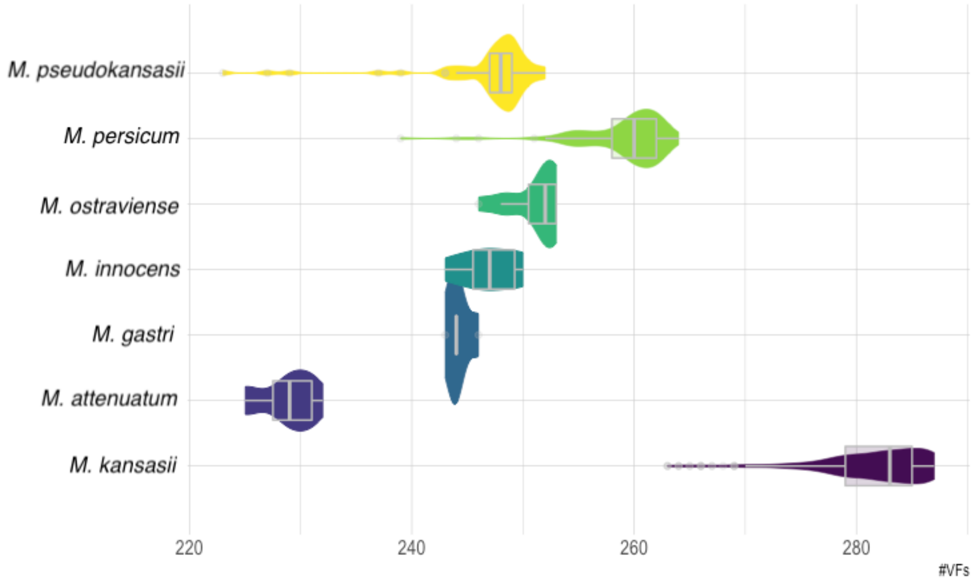

Supplementary Figure 5. **MKC virulome characteristics**. Violin plot showing the number of virulence factors (x axis) for each MKC species (y axis). Density peaks of virulence factors inferred are shown in the graph for each MKC species. Additionally, internal box plots shows summary statistics (median and interquartile). The thin gray line represents the rest of the distribution, whereas points are outliers.

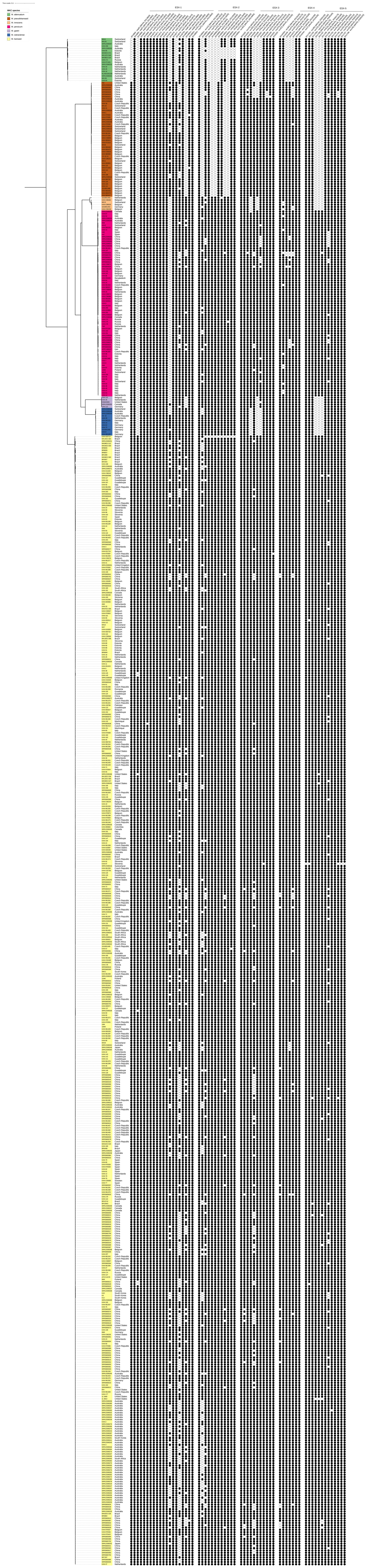
Tree scale: 0.1
MKC species
M. attenuatum
M. pseudokansasii
M. innocens
M. persicum
M. gastri
M. ostraviense
M. kansasii
ESX-1
ESX-2
ESX-3
ESX-4
ESX-5
(Rv3864 - espE) [MKAN_RS13800]
(Rv3865 - espF) [MKAN_RS13805]
(Rv3866 - espG1) [MKAN_RS07265]
(Rv3867 - espH) [MKAN_RS13815]
(Rv3868 - eccA1) [MKAN_RS13820]
(Rv3869 - eccB1) [MKAN_RS13825]
(Rv3870 - eccCa1) [MKAN_RS13830]
(Rv3871 - eccCb1) [MKAN_RS13835]
(Rv3872 - PE35) [MKAN_RS13840]
(Rv3873 - PPE68) [MKAN_RS13845]
(Rv3874 - esxB) [MKAN_RS13850]
(Rv3875 - esxA) [MKAN_RS13855]
(Rv3876 - espI) [MKAN_RS29685]
(Rv3877 - eccD1) [MKAN_RS13870]
(Rv3878 - espJ) [MKAN_RS13875]
(Rv3879c - espK) [MKAN_RS13880]
(Rv3880c - espL) [MKAN_RS13885]
(Rv3881c - espB) [MKAN_RS13890]
(Rv3882c - eccE1) [MKAN_RS13895]
(Rv3883c - mycP1) [MKAN_RS13900]
(Rv3884c - eccA2) [MKAN_RS13905]
(Rv3885c - eccE2) [MKAN_RS13910]
(Rv3886c - mycP2) [MKAN_RS13915]
(Rv3887c - eccD2) [MKAN_RS13920]
(Rv3888c) [MKAN_RS13925]
(Rv3889c - espG2) [MKAN_RS13930]
(Rv3890c - esxC) [MKAN_RS13935]
(Rv3891c - esxD) [MKAN_RS13940]
(Rv3892c - PPE69) [MKAN_RS13945]
(Rv3893c - PE36) [MKAN_RS13950]
(Rv3894c - eccC2) [MKAN_RS13960]
(Rv3895c - eccB2) [MKAN_RS13965]
(Rv0282 - eccA3) [MKAN_RS16180]
(Rv0283 - eccB3) [MKAN_RS16185]
(Rv0284 - eccC3) [MKAN_RS16190]
(Rv0285 - PE5) [MKAN_RS16160]
(Rv0286 - PPE4) [MKAN_RS16200]
(Rv0287 - esxG) [MKAN_RS06755]
(Rv0288 - esxH) [MKAN_RS19710]
(Rv0289 - espG3) [MKAN_RS16215]
(Rv0290 - eccD3) [MKAN_RS16220]
(Rv0291 - mycP3) [MKAN_RS16225]
(Rv0292 - eccE3) [MKAN_RS16230]
(Rv3450c - eccB4) [MKAN_RS19165]
(Rv3449 - mycP4) [MKAN_RS19170]
(Rv3448 - eccD4) [MKAN_RS19175]
(Rv3447c - eccC4) [MKAN_RS19180]
(Rv3446c) [MKAN_RS19185]
(Rv3445c - esxU) [MKAN_RS19190]
(Rv3444c - esxT) [MKAN_RS19195]
(Rv1782 - eccB5) [MKAN_RS00155]
(Rv1783 - eccC5) [MKAN_RS00160]
(Rv1785c - cyp143) [MKAN_RS00165]
(Rv1786) [MKAN_RS00170]
(Rv1787 - PPE25) [MKAN_RS00175]
(Rv1789 - PPE26) [MKAN_RS00185]
(Rv1788 - PE18) [MKAN_RS00190]
(Rv1793 - esxN) [MKAN_RS00200]
(Rv1794 - espG5) [MKAN_RS00205]
(Rv1795 - eccD5) [MKAN_RS00210]
(Rv1796 - mycP5) [MKAN_RS00215]
(Rv1797 - eccE5) [MKAN_RS00220]
(Rv1798 - eccA5) [MKAN_RS00225]

Supplementary Figure 6. **Presence or absence of the Type VII secretion system (T7SS) genes among *M. kansasii* complex genomes.** Midpoint rooted maximum likelihood phylogenetic tree of 665 *M. kansasii* complex genomes and the presence or absence of the Type VII secretion system (T7SS) genes. Node names are colored by MKC species, as indicated in the left upper legend. Genes are organized in the five paralogous ESX loci (ESX-1 to ESX-5), with the reference *M. kansasii* ATCC12478 locus name in brackets. Additionally, the respective *M. tuberculosis* H37Rv orthologous gene name and locus are given in parenthesis. Circles represents the presence (black filled) or absence (unfilled).

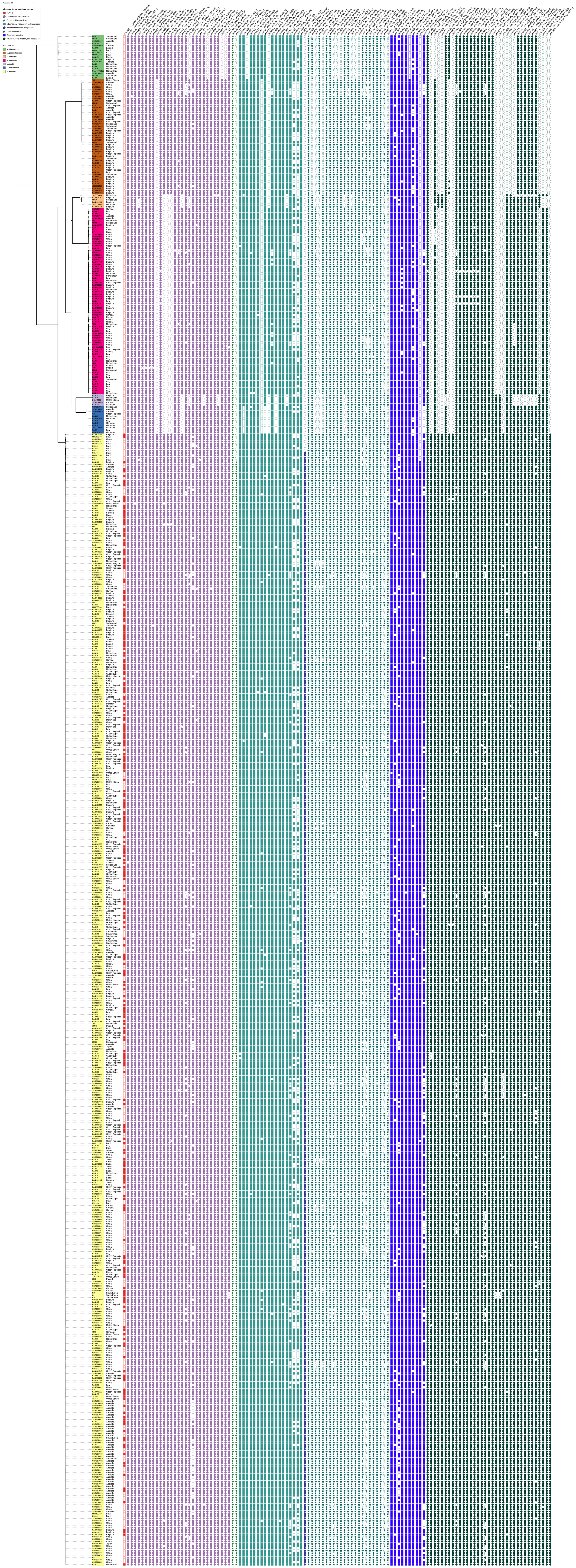
Tree scale: 0.1
Virulence factor functional category
PE/PPE
Cell wall and cell processes
Conserved hypotheticals
Intermediary metabolism and respiration
Insertion sequences and phages
Lipid metabolism
Regulatory proteins
Virulence, detoxification, and adaptation
MKC species
M. attenuatum
M. pseudokansasii
M. innocens
M. persicum
M. gastri
M. ostraviense
M. kansasii

Supplementary Figure 7. **Presence or absence of 119 virulence factors among *M. kansasii* complex genomes**. Midpoint rooted maximum likelihood phylogenetic tree of 665 M. kansasii complex genomes and the presence or absence of 119 virulence factors (VFs). Node names are colored by MKC species, as indicated in the left upper legend. The presence (filled shapes) or absence (unfilled shapes) of VFs for each genome is indicated. Functional category of VFs is indicated by shape forms and colors. The reference *M. kansasii* ATCC12478 locus name is given in brackets, whereas the respective *M. tuberculosis* H37Rv orthologous gene name and locus are given in parenthesis.

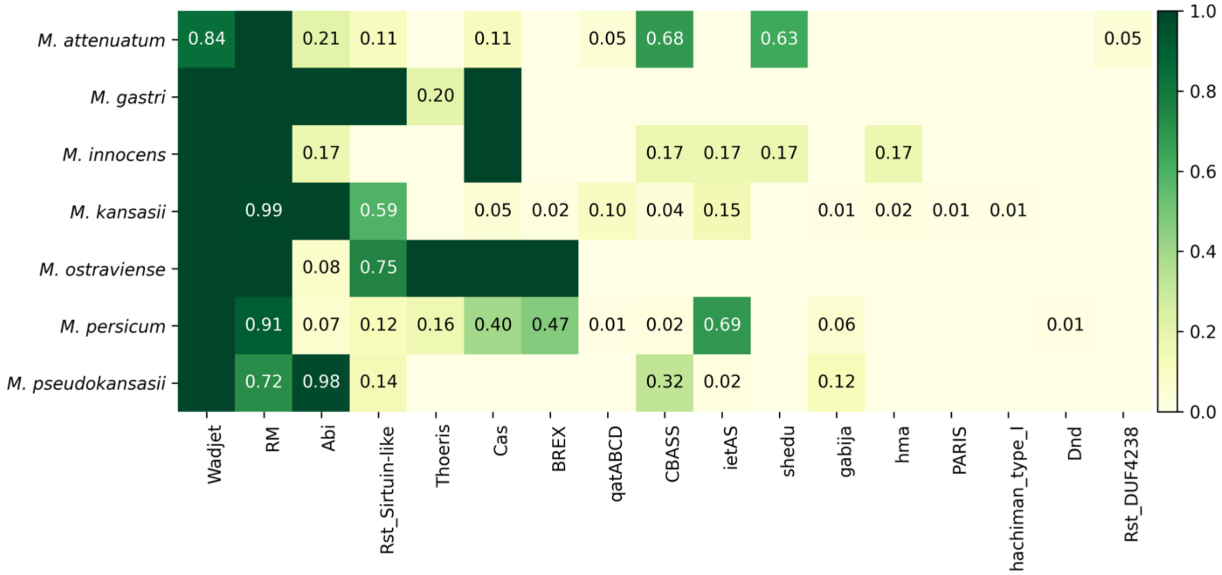

Supplementary Figure 8. MKC defense systems. The heatmap shows defense systems (horizontal axis) found in MKC species (vertical axis) and their proportion per species. Cells with suppressed values, indicate systems either present (dark colors) or absent (light colors) in all genomes of a given species. Numbers in cells specify the proportion of systems found only in part of genomes of a given species, with light colors representing low proportions and darker colors high proportions.

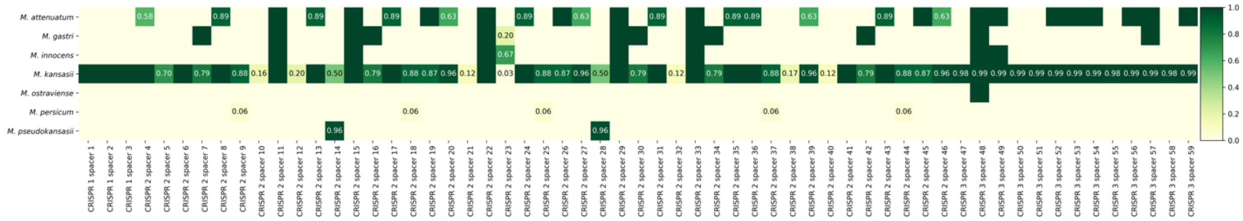

Supplementary Figure 9. ***M. kansasii* CRISPR loci.** The presence or absence of CRISPR spacers (horizontal) among MKC species (vertical) is represented in a heatmap. The 59 spacers belong to three CRISPR loci found in genome of the *M. kansasii* ATCC 12478 strain. Spacers either totally present (dark colors) or totally absent (light colors) in all genomes of a given species are indicated in cells with suppressed values. Alternatively, the proportion of spacers found partially in each species is explicit indicated in the respective cells, as well represented by color degree, with light for low and darker for high proportions.

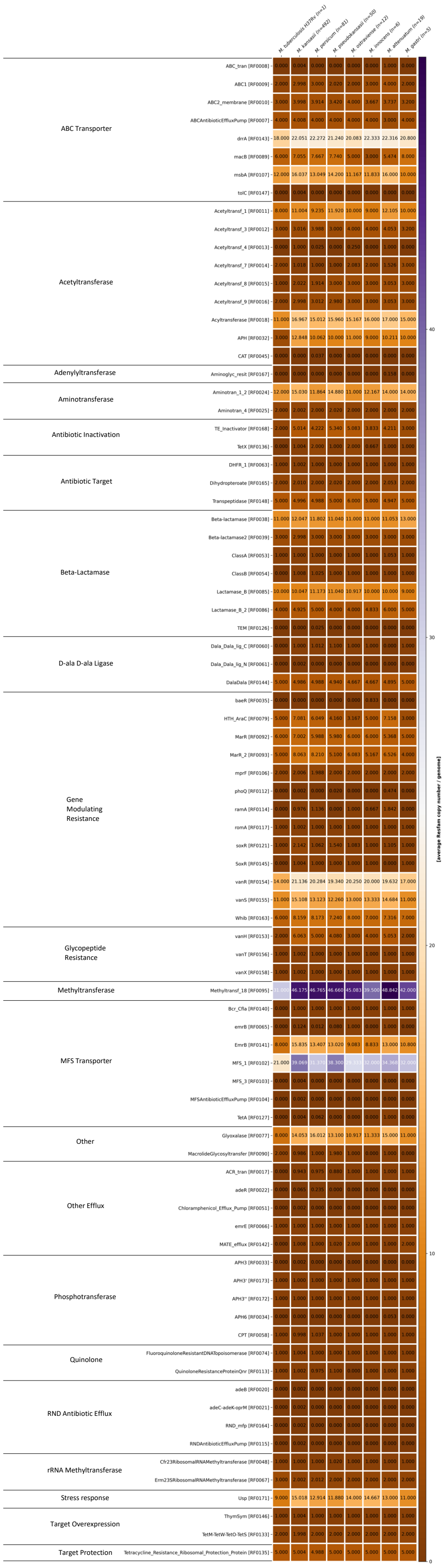

M. tuberculosis H37Rv (n=1)
M. kansasii (n=492)
M. persicum (n=81)
M. pseudokansasii (n=50)
M. ostraviense (n=12)
M. innocens (n=6)
M. attenuatum (n=19)
M. gastri (n=5)
ABC Transporter
ABC_tran [RF0008] 0.000 0.004 0.000 0.000 0.000 0.000 1.000 0.000
ABC1 [RF0009] 2.000 2.998 3.000 2.020 2.000 3.000 4.000 2.000
ABC2_membrane [RF0010] 3.000 3.998 3.914 3.420 4.000 3.667 3.737 3.200
ABCAntibioticEffluxPump [RF0007] 4.000 4.008 4.000 4.000 4.000 4.000 3.000 4.000
drrA [RF0143] 18.000 22.051 22.272 21.240 20.083 22.333 22.316 20.800
macB [RF0089] 6.000 7.055 7.667 7.740 5.000 3.000 5.474 8.000
msbA [RF0107] 12.000 16.037 13.049 14.200 11.167 11.833 16.000 10.000
tolC [RF0147] 0.000 0.004 0.000 0.000 0.000 0.000 0.000 0.000
Acetyltransferase
Acetyltransf_1 [RF0011] 8.000 11.004 9.235 11.920 10.000 9.000 12.105 10.000
Acetyltransf_3 [RF0012] 3.000 3.016 3.988 3.000 4.000 4.000 4.053 3.200
Acetyltransf_4 [RF0013] 0.000 1.000 0.025 0.000 0.250 0.000 1.000 0.000
Acetyltransf_7 [RF0014] 2.000 1.018 1.000 1.000 2.083 2.000 1.526 3.000
Acetyltransf_8 [RF0015] 1.000 2.022 1.914 3.000 3.000 3.000 3.053 3.000
Acetyltransf_9 [RF0016] 2.000 2.998 3.012 2.980 3.000 3.000 3.053 3.000
Acyltransferase [RF0018] 11.000 16.967 15.012 15.960 15.167 16.000 17.000 15.000
APH [RF0032] 3.000 12.848 10.062 10.000 11.000 9.000 10.211 10.000
CAT [RF0045] 0.000 0.000 0.037 0.000 0.000 0.000 0.000 0.000
Adenylyltransferase
Aminoglyc_resit [RF0167] 0.000 0.000 0.000 0.000 0.000 0.000 0.158 0.000
Aminotransferase
Aminotran_1_2 [RF0024] 12.000 15.030 11.864 14.880 11.000 12.167 14.000 14.000
Aminotran_4 [RF0025] 2.000 2.002 2.000 2.020 2.000 2.000 2.000 2.000
Antibiotic Inactivation
TE_Inactivator [RF0168] 2.000 5.014 4.222 5.340 5.083 3.833 4.211 3.000
TetX [RF0136] 0.000 1.004 2.000 1.000 2.000 0.667 1.000 1.000
Antibiotic Target
DHFR_1 [RF0063] 1.000 1.002 1.000 1.000 1.000 1.000 1.000 1.000
Dihydropteroate [RF0165] 2.000 2.010 2.000 2.020 2.000 2.000 2.053 2.000
Transpeptidase [RF0148] 5.000 4.996 4.988 5.000 6.000 5.000 4.947 5.000
Beta-Lactamase
Beta-lactamase [RF0038] 11.000 12.047 11.802 11.040 11.000 11.000 11.053 13.000
Beta-lactamase2 [RF0039] 3.000 2.998 3.000 3.000 3.000 3.000 3.000 3.000
ClassA [RF0053] 1.000 1.000 1.000 1.000 1.000 1.000 1.053 1.000
ClassB [RF0054] 0.000 1.008 1.025 1.000 1.000 1.000 1.000 1.000
Lactamase_B [RF0085] 10.000 10.047 11.173 11.040 10.917 10.000 10.000 9.000
Lactamase_B_2 [RF0086] 4.000 4.925 5.000 4.000 4.000 4.833 6.000 5.000
TEM [RF0126] 0.000 0.000 0.025 0.000 0.000 0.000 0.000 0.000
D-ala D-ala Ligase
Dala_Dala_lig_C [RF0060] 0.000 1.000 1.012 1.100 1.000 1.000 0.000 1.000
Dala_Dala_lig_N [RF0061] 0.000 0.002 0.000 0.000 0.000 0.000 0.000 0.000
DalaDala [RF0144] 5.000 4.986 4.988 4.940 4.667 4.667 4.895 5.000
Gene Modulating Resistance
baeR [RF0035] 0.000 0.000 0.000 0.000 0.000 0.833 0.000 0.000
HTH_AraC [RF0079] 5.000 7.081 6.049 4.160 3.167 5.000 7.158 3.000
MarR [RF0092] 6.000 7.002 5.988 5.980 6.000 6.000 5.368 5.000
MarR_2 [RF0093] 5.000 8.063 8.210 5.100 6.083 5.167 6.526 4.000
mprF [RF0106] 2.000 2.006 1.988 2.000 2.000 2.000 2.000 2.000
phoQ [RF0112] 0.000 0.002 0.000 0.020 0.000 0.000 0.474 0.000
ramA [RF0114] 0.000 0.976 1.136 0.000 1.000 0.667 1.842 0.000
romA [RF0117] 1.000 1.002 1.000 1.000 1.000 1.000 1.000 1.000
soxR [RF0121] 1.000 2.142 1.062 1.540 1.083 1.000 1.105 1.000
SoxR [RF0145] 0.000 1.004 1.000 1.000 1.000 1.000 0.000 1.000
vanR [RF0154] 14.000 21.136 20.284 19.340 20.250 20.000 19.632 17.000
vanS [RF0155] 11.000 15.108 13.123 12.260 13.000 13.333 14.684 11.000
Whib [RF0163] 6.000 8.159 8.173 7.240 8.000 7.000 7.316 7.000
Glycopeptide Resistance
vanH [RF0153] 2.000 6.063 5.000 4.080 3.000 4.000 5.053 2.000
vanT [RF0156] 1.000 1.002 1.000 1.000 1.000 1.000 1.000 1.000
vanX [RF0158] 1.000 1.002 1.000 1.000 1.000 1.000 1.000 1.000
Methyltransferase
Methyltransf_18 [RF0095] 31.000 46.175 46.765 46.660 45.083 39.500 48.842 42.000
MFS Transporter
Bcr_Cfla [RF0140] 0.000 1.000 1.000 1.000 1.000 1.000 1.000 1.000
emrB [RF0065] 0.000 0.124 0.012 0.080 1.000 0.000 1.000 0.000
EmrB [RF0141] 8.000 15.835 13.407 13.020 9.083 8.833 13.000 10.800
MFS_1 [RF0102] 21.000 39.069 31.370 38.300 29.333 32.000 34.368 32.000
MFS_3 [RF0103] 0.000 0.004 0.000 0.000 0.000 0.000 0.000 0.000
MFSAntibioticEffluxPump [RF0104] 0.000 0.002 0.000 0.000 0.000 0.000 0.000 0.000
TetA [RF0127] 0.000 0.004 0.062 0.000 0.000 0.000 1.000 0.000
Other
Glyoxalase [RF0077] 8.000 14.053 16.012 13.100 10.917 11.333 15.000 11.000
MacrolideGlycosyltransfer [RF0090] 2.000 0.986 1.000 1.980 1.000 0.000 1.000 0.000
Other Efflux
ACR_tran [RF0017] 0.000 0.943 0.975 0.880 1.000 1.000 1.000 1.000
adeR [RF0022] 0.000 0.065 0.235 0.000 0.000 0.000 0.000 0.000
Chloramphenicol_Efflux_Pump [RF0051] 0.000 0.002 0.000 0.000 0.000 0.000 0.000 0.000
emrE [RF0066] 1.000 1.000 1.000 1.000 1.000 1.000 1.000 1.000
MATE_efflux [RF0142] 0.000 1.008 1.000 1.020 2.000 1.000 1.000 2.000
Phosphotransferase
APH3 [RF0033] 0.000 0.002 0.000 0.000 0.000 0.000 0.000 0.000
APH3' [RF0173] 1.000 1.000 1.000 1.000 1.000 1.000 1.000 1.000
APH3'' [RF0172] 1.000 1.000 1.000 1.000 1.000 1.000 1.000 1.000
APH6 [RF0034] 0.000 0.000 0.000 0.000 0.000 0.000 0.053 0.000
CPT [RF0058] 1.000 0.998 1.037 1.000 1.000 1.000 1.000 1.000
Quinolone
FluoroquinoloneResistantDNATopoisomerase [RF0074] 1.000 1.004 1.000 1.000 1.000 1.000 1.000 1.000
QuinoloneResistanceProteinQnr [RF0113] 1.000 1.002 0.975 1.100 0.000 1.000 1.000 1.000
RND Antibiotic Efflux
adeB [RF0020] 0.000 0.002 0.000 0.000 0.000 0.000 0.000 0.000
adeC-adeK-oprM [RF0021] 0.000 0.002 0.000 0.000 0.000 0.000 0.000 0.000
RND_mfp [RF0164] 0.000 0.002 0.000 0.000 0.000 0.000 0.000 0.000
RNDAntibioticEffluxPump [RF0115] 0.000 0.002 0.000 0.000 0.000 0.000 0.000 0.000
rRNA Methyltransferase
Cfr23RibosomalRNAMethyltransferase [RF0048] 1.000 1.000 1.000 1.020 1.000 1.000 1.000 1.000
Erm23SRibosomalRNAMethyltransferase [RF0067] 3.000 2.002 2.012 2.000 2.000 2.000 2.000 2.000
Stress response
Usp [RF0171] 9.000 15.018 12.914 11.880 14.000 14.667 13.000 11.000
Target Overexpression
ThymSym [RF0146] 1.000 1.004 1.000 1.000 1.000 1.000 1.000 1.000
TetM-TetW-TetO-TetS [RF0133] 2.000 1.998 2.000 2.000 2.000 2.000 2.000 2.000
Target Protection
Tetracycline_Resistance_Ribosomal_Protection_Protein [RF0135] 5.000 5.004 4.988 5.000 5.000 5.000 5.000 5.000
0
10
20
30
40
[average Resfam copy number / genome]

Supplementary Figure 10. **MKC resistome analysis.** Heatmaps show the average copy number of resistance protein profiles (Resfams) among each MKC species' genomes in comparison with *M. tuberculosis* H37Rv. The graphs show 83 Resfams (second column), classified into 21 resistance mechanisms (first column), found in all MKC species. The average number of copies per genome per species is indicated inside the cells and represented by color degree, according to the legend on the right side of each graph.

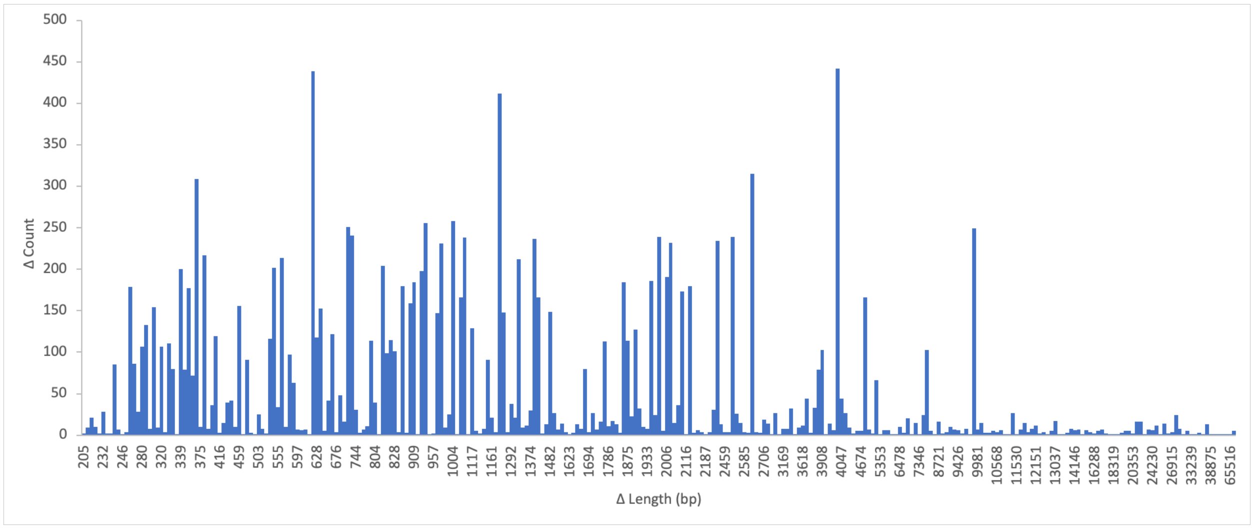

Supplementary Figure 11. Characteristics of regions of difference in *M. kansasii* genomes. Size distribution of regions of difference among *M. kansasii* genomes.

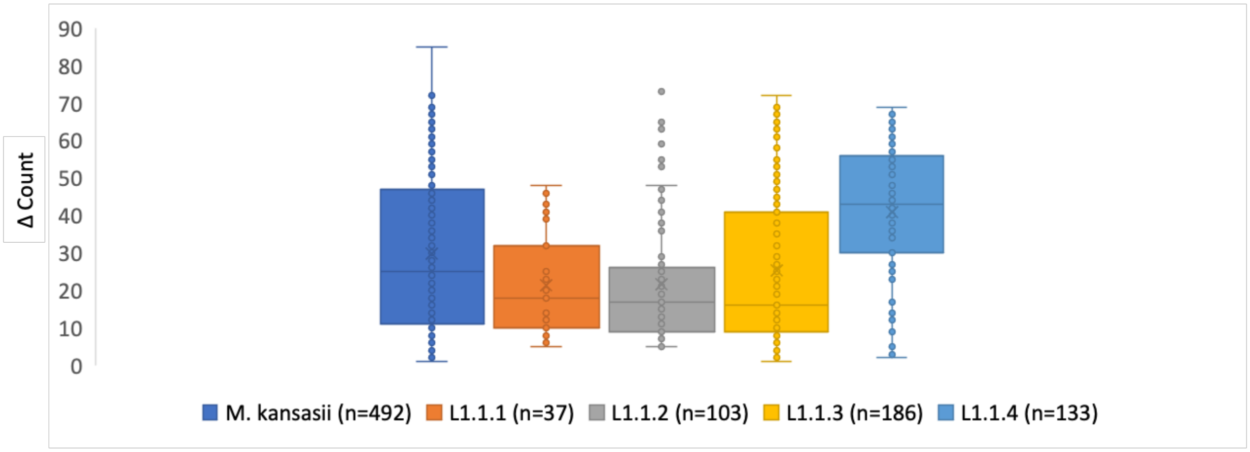

Supplementary Figure 12. Distribution of regions of difference by *M. kansasii* sub-lineage. Regions of difference per genome distribution among genomes of the *M. kansasii* sub-lineages. Each point represents an individual sample. The number of regions per genome is indicated in the y axis. Boxes represent the interquartile range with 50% of the values. The median is indicated by a line across the box and the mean by a "X".
